# Vm–MSI: a Vancomycin–Antimicrobial Peptide Conjugate Combating Resistant Bacteria and Broadening the Antimicrobial Spectrum

**DOI:** 10.1002/advs.202503023

**Published:** 2025-12-08

**Authors:** Shuangyu Li, Kang Wang, Wenzhuang Shi, Xu Wang, Duxin Li, Yanli Liu, Peng Zhang, Yipeng Wang

**Affiliations:** ^1^ Yantai Institute of Coastal Zone Research Chinese Academy of Sciences Yantai Shandong 264003 China; ^2^ Department of Biopharmaceutical Sciences College of Pharmaceutical Sciences Soochow University Suzhou Jiangsu 215123 China; ^3^ University of Chinese Academy of Sciences Beijing 100049 China; ^4^ Department of Orthopaedics The Second Affiliated Hospital of Soochow University Suzhou Jiangsu 215004 China

**Keywords:** antimicrobial peptide, antimicrobial spectrum, bacterial resistance, structural modification, vancomycin

## Abstract

Vancomycin is a critical last‐resort treatment for multidrug‐resistant Gram‐positive bacteria, particularly severe methicillin‐resistant *S. aureus* (MRSA) infections. However, the rise of vancomycin‐resistant strains significantly compromises its therapeutic efficacy. To overcome this challenge, recent research has focused on structural modifications of vancomycin using diverse strategies. Herein, the potential of a modification strategy by coupling vancomycin with antimicrobial peptides (AMPs) that have different mechanisms of action is explored. Among the acquired conjugates, Vm‐MSI showed a 20.18 fold improvement in antimicrobial activity over vancomycin and a 1.95 fold increase compared to the parent peptide MSI‐78. Vm‐MSI not only delays the development of resistance in vancomycin‐resistant *S. aureus* (VRSA) but also exhibits potent activity against a wide range of Gram‐negative bacteria. Additionally, Vm‐MSI demonstrated strong synergy with several conventional antibiotics of distinct mechanisms and displayed potent activities in eradicating biofilms and persisters. Mechanistic studies revealed the complex antibacterial mechanisms of Vm‐MSI, which hinder the development of bacterial resistance. In vivo, Vm‐MSI displayed remarkable therapeutic efficacy in mouse models of VRSA‐induced skin infection and multidrug‐resistant *A. baumannii*‐induced lung infection. These findings underscore the great potential of Vm‐MSI as an effective treatment for infections caused by vancomycin‐resistant and Gram‐negative bacteria.

## Graphical abstract

A series of vancomycin‐antimicrobial peptide conjugates is synthesized and evaluated to identify the optimal combination. Vm‐MSI, selected from multiple candidates, exhibited potent activity against vancomycin‐resistant and Gram‐negative bacteria by disrupting membranes and inducing oxidative stress, thereby expanding vancomycin's antibacterial spectrum and delaying resistance development.

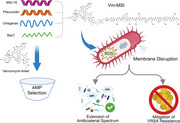

## 1 Introduction

The rapid rise of bacterial resistance in recent years poses a significant threat to global health. Antibiotic resistance has become the third leading cause of death worldwide, with projections estimating that by 2050, over 10 million people will die annually from infections caused by drug‐resistant bacteria.^[^ ^1^ ^]^ The ESKAPE group (*E. faecalis*, *S. aureus*, *K. pneumoniae*, *A. baumannii*, *P. aeruginosa*, and *Enterobacter*) has emerged as the leading group of multidrug‐resistant bacteria in clinical settings, characterized by its extensive repertoire of drug‐resistant genes and strong environmental resilience.^[^ ^2^ ^]^ Thus, there is an urgent need to develop novel antimicrobial agents that are effective against drug‐resistant pathogens.

Since its discovery in 1952, vancomycin has been widely used to treat severe infections caused by MRSA and other Gram‐positive bacteria. Its antimicrobial mechanism involves inhibiting cell wall synthesis by binding to the “D‐Ala‐D‐Ala” sequence in the peptidoglycan precursor within the bacterial cell wall.^[^ ^3^ ^]^ Unfortunately, despite being a last‐resort treatment for severe Gram‐positive infections, vancomycin has not been able to prevent the emergence of bacterial resistance. Among the 18 bacterial and fungal pathogens highlighted in the 2019 Antimicrobial Resistance Threat Report, VRSA stands out as a critical concern.^[^ ^4^ ^]^ In VRSA, mutations in the target site alter the “D‐Ala‐D‐Ala” sequence to “D‐Ala‐D‐Ser” or “D‐Ala‐D‐Lac”. This modification reduces vancomycin's binding efficacy, significantly compromising its antibacterial potency.^[^ ^5^, ^6^ ^]^ Additionally, bacterial cell wall thickening contributes to VRSA resistance by creating a spatial barrier that hinders vancomycin's access to its target sites within the cytoplasmic membrane.^[^ ^7^, ^8^ ^]^ Moreover, Gram‐negative bacteria are inherently resistant to vancomycin due to their dense outer membrane, which blocks the drug's penetration. This structural barrier in Gram‐negative bacteria significantly limits vancomycin's antimicrobial spectrum.^[^ ^9^, ^10^ ^]^

AMPs are naturally occurring bioactive peptides found in animals, plants, and microorganisms.^[^ ^11^ ^]^ Most AMPs exert their bactericidal effects by disrupting the integrity of bacterial cell membranes through three primary models: the barrel‐stave, carpet, and toroidal‐pore models.^[^ ^11^, ^12^ ^]^ This disruption causes the release of intracellular contents, leading to rapid bacterial death. Additionally, many AMPs can translocate across the cell membrane and inhibit the biosynthesis of key bacterial biomolecules such as DNA, RNA, and proteins.^[^ ^13^, ^14^, ^15^ ^]^ The diverse antimicrobial mechanisms of AMPs are closely related to their amino acid composition, hydrophobicity, charge distribution, sequence length, and secondary structure. Unlike conventional small‐molecule antibiotics, AMPs lack a single binding target, making it more difficult for bacteria to develop resistance.^[^ ^13^, ^14^ ^]^ As a result, AMPs are widely regarded as highly promising and innovative antibacterial agents in the post‐antibiotic era. To date, over 30 AMPs have been extensively studied in clinical research, demonstrating remarkable therapeutic efficacy in treating conditions such as diabetic foot ulcers, rosacea, acne, and sepsis.^[^ ^12^ ^]^

In recent years, structural modifications of vancomycin have become a key area of research aimed at overcoming bacterial resistance and restoring its antibacterial activity. Research on antibiotic structural modification has concentrated on two primary approaches: first, improving vancomycin's binding affinity to peptidoglycan by incorporating hydrophilic groups, such as glycosyl groups;^[^ ^16^, ^17^ ^]^ and second, introducing cationic and hydrophobic groups to engage new target sites or mechanisms.^[^ ^18^, ^19^, ^20^, ^21^ ^]^ A recent innovative approach involves modifying vancomycin through peptide engineering. By incorporating polycationic peptides and hydrophobic moieties, vancomycin resistance in *E. faecalis* can be effectively circumvented, while also enhancing its antibacterial efficacy against susceptible bacteria.^[^ ^22^, ^23^ ^]^ Furthermore, incorporating the immunomodulatory host defense peptide (HDP) IDR‐1018, which lacks direct antibacterial activity, adds additional functionalities to vancomycin, including anti‐biofilm properties and immune regulation.^[^ ^18^ ^]^ However, challenges such as the emergence of drug resistance, a narrow antibacterial spectrum, and unexpected toxicity remain with these vancomycin derivatives. Given the numerous advantages and diverse mechanisms of AMPs, utilizing them for vancomycin modification could be a promising strategy. Although previous studies have explored AMP–vancomycin conjugates, systematic evaluation of AMP class selection, structure–activity relationship, and spectrum‐expanding potential remains limited.

In this study, we selected four AMPs with distinct mechanisms of action and conjugated them with vancomycin, aiming to generate multifunctional conjugates. By anchoring AMPs to vancomycin, we evaluated their biological activities and therapeutic performance both in vitro and in vivo. Among the resulting conjugates, the lead compound, Vm‐MSI, demonstrated significantly enhanced antimicrobial activity compared to vancomycin and the parent peptide MSI‐78. Vm‐MSI delayed the development of vancomycin resistance in VRSA and exhibited broad‐spectrum efficacy against both standard and drug‐resistant Gram‐positive and Gram‐negative bacteria. It also showed strong synergy with multiple conventional antibiotics, displayed potent anti‐biofilm activity, and effectively eliminated persister cells. Mechanistic studies revealed that Vm‐MSI employs a multifaceted bacterial eradication strategy, including membrane binding and disruption, retention of vancomycin's binding to the “D‐Ala‐D‐Ala” target sequence, thereby inhibiting cell wall synthesis, and promotion of ROS production leading to oxidative damage. These combined mechanisms help reduce the likelihood of bacterial resistance development. Finally, animal studies demonstrated the exceptional therapeutic efficacy of Vm‐MSI in both a VRSA‐induced mouse skin infection model and a multidrug‐resistant Gram‐negative *A. baumannii*‐induced mouse lung infection model.

## 2 Results

### 2.1 Design, Characterization, and Screening of Vancomycin‐AMP Conjugates

To facilitate the structural modification of vancomycin, we employed coupling strategies that combined vancomycin with AMPs. Four AMPs with distinct mechanisms of action were selected for this purpose (Table S1, Supporting Information). One of these, omiganan, is a derivative of indolicidin, a peptide secreted by bovine neutrophils. Omiganan exerts bactericidal effects by inhibiting bacterial DNA and RNA synthesis and inducing membrane depolarization. Currently, it is undergoing Phase II clinical trials for the treatment of seborrheic dermatitis on the face.^[^ ^24^, ^25^ ^]^ MSI‐78, a derivative of magainin II isolated from the skin secretion of the African clawed frog, exhibits bactericidal activity by disrupting bacterial cell membrane integrity. It has advanced to Phase III clinical trials as an active ingredient in a topical cream for treating diabetic foot infections, although it has not received FDA approval.^[^ ^26^ ^]^ Bac‐7, an AMP derived from the cow *Bos taurus*, specifically binds to the exit tunnel of bacterial ribosomes, inhibiting the elongation phase of protein translation.^[^ ^27^, ^28^, ^29^ ^]^ Pleurocidin, an AMP derived from the fish *Pleuronectes americanus*, exerts bactericidal effects through inhibition of protein synthesis as well as induction of cell membrane depolarization.^[^ ^30^ ^]^

To facilitate the conjugation, a cysteine residue was introduced at the N‐terminal end of the native AMP sequence. The succinimidyl ester of sulfo‐SMCC specifically reacts with the secondary amine moiety of vancomycin, forming an amide bond. The maleimide group at the opposite end of sulfo‐SMCC then forms a stable covalent bond with the sulfhydryl group of the N‐terminal cysteine residue in the AMPs.^[^ ^22^ ^]^ The successful synthesis of all target products was confirmed by high‐performance liquid chromatography (HPLC) and liquid chromatography‐mass spectrometry (LC‐MS) analysis (Figures S1 and S2, Supporting Information), resulting in the identification of four vancomycin‐AMP conjugates: Vm‐Omi, Vm‐MSI, Vm‐Ple, and Vm‐Bac.

To identify a lead compound, we evaluated the minimum inhibitory concentrations (MICs) of the four conjugates against 13 bacterial strains, including both standard and drug‐resistant Gram‐positive and Gram‐negative bacteria. The results showed that Vm‐Omi exhibited antimicrobial activity exclusively against *S. aureus*, while Vm‐MSI, Vm‐Ple, and Vm‐Bac demonstrated robust and broad‐spectrum activity (Table S2, Supporting Information). The geometric mean (GM) MIC values for Vm‐MSI, Vm‐Ple, and Vm‐Bac were 2.60, 4.67, and 6.64 µm, respectively, with lower GM values indicating higher antimicrobial activity. Among the conjugates, Vm‐MSI showed the most potent activity. Notably, Vm‐MSI effectively delays the development of resistance in VRSA and exhibits strong antibacterial efficacy against Gram‐negative bacteria, which are typically inherently resistant to vancomycin. Importantly, Vm‐MSI displayed the greatest enhancement in antimicrobial activity compared to both vancomycin and the parent peptide. To investigate whether vancomycin conjugation altered the secondary structure of MSI‐78, circular dichroism (CD) spectroscopy was performed. In aqueous solution, both MSI‐78 and Vm‐MSI exhibited disordered conformations with negligible α‐helical characteristics. In contrast, under membrane‐mimicking conditions (60 mm SDS), MSI‐78 displayed a typical α‐helical spectrum with negative peaks at 208 and 222 nm, whereas Vm‐MSI showed significantly attenuated helical signals at the same wavelengths. These results suggest that the covalent attachment of vancomycin interferes with the helical folding of MSI‐78 (Figure S3, Supporting Information), potentially altering its membrane interaction behavior and biological function.

The role of bacterial biofilms as a key contributor to bacterial resistance is well‐established.^[^ ^20^ ^]^ Anti‐biofilm activity is a crucial evaluation criterion for novel antimicrobial drugs. Therefore, we assessed the anti‐biofilm activity of the four conjugates against *S. aureus* CMCC26003 and *E. coli* ATCC25922 (Figure S4, Supporting Information). The conjugates showed notable concentration‐dependent biofilm inhibition and preformed biofilm eradication effects, with Vm‐MSI demonstrating exceptional biofilm‐scavenging activity at a low concentration of 1 µm. At a concentration of 4 µm, Vm‐MSI inhibited the formation of 80% of *S. aureus* biofilms and 60% of *E. coli* biofilms.

**Figure 1:**
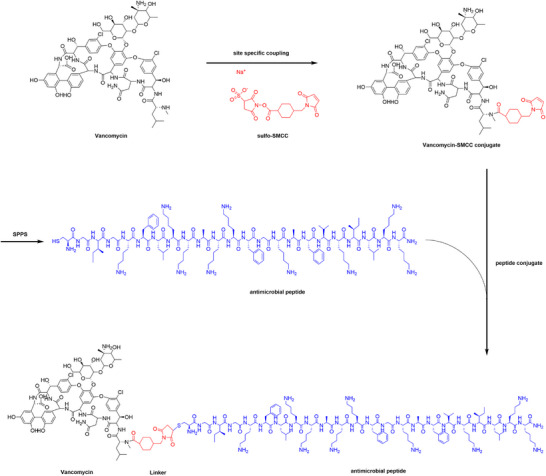
Synthesis route of vancomycin‐AMP conjugate. The black structure represents vancomycin, the red structure corresponds to the linker, and the blue structure denotes AMP. Vancomycin was first stirred with Sulfo‐SMCC at a 1:1 molar ratio at room temperature for 3 h, followed by reaction with AMP in PBS at a 1:1 molar ratio at room temperature for 2 h with stirring.

To assess the biosafety of the conjugates, a hemolysis assay was performed on both fresh human and murine blood cells for all four candidate conjugates (Figure S5, Supporting Information). All conjugates exhibited minimal hemolysis within the effective antimicrobial concentration range, with Vm‐MSI showing only 10% hemolysis toward human blood cells at 32 µm (≈12 times the GM value). Given its potent antimicrobial activity, strong anti‐biofilm efficacy, and excellent biosafety profile, Vm‐MSI was identified as the optimal lead candidate for further investigation (**Figure** **1**).

### 2.2 In Vitro Antimicrobial Activity of Vm‐MSI

To further assess the antimicrobial activity and antibacterial spectrum of Vm‐MSI, we conducted a comprehensive evaluation of its efficacy against 29 standard and multidrug‐resistant bacterial strains, including 11 Gram‐positive bacteria and 18 Gram‐negative bacteria. The GM values revealed that Vm‐MSI exhibited significantly greater antibacterial activity than vancomycin alone, with a 20.18 fold increase in potency. Additionally, it outperformed MSI‐78 by 1.95 fold and the physically mixed combination of vancomycin and MSI‐78 by 2.56 fold (**Table** **1**). Notably, due to assay concentration limitations, MIC values for vancomycin exceeding 100 µg mL^−1^ were defaulted to 100 µg mL^−1^ in the GM value calculation. Therefore, the actual antimicrobial activity of Vm‐MSI significantly surpasses the 20.18 fold improvement over vancomycin. Specifically, Vm‐MSI not only exhibits potent activity against VRSA but also exhibits strong antimicrobial activity against a broad range of Gram‐negative bacteria. Multidrug‐resistant Gram‐negative strains such as MDR *A. baumannii*, MDR *P. aeruginosa*, and MDR *K. pneumoniae*, the primary pathogens responsible for clinically refractory infections, were highly susceptible to Vm‐MSI. The MIC values for these pathogens ranged from 1.10 to 4.42 µm, highlighting Vm‐MSI's efficacy as a potent agent against Gram‐negative multidrug‐resistant pathogens.

**Table 1:** Antimicrobial activity of vancomycin, MSI‐78, Vm‐MSI, and the physically mixed combination of vancomycin and MSI‐78.

MIC^a)^ µm (µg mL^−1^)				
Bacteria	Vm	MSI‐78	Vm+MSI‐78^b)^	Vm‐MSI
Gram‐positive				
*S. aureus* CMCC26003	0.40(0.59)	0.94(2.34)	0.32(0.59)	0.28(1.17)
*S. aureus* ATCC29213	3.16(4.69)	15.14(37.5)	2.53(4.69)	0.55(2.34)
MRSA ATCC43300	3.16(4.69)	1.89(4.69)	2.53(4.69)	1.10(4.69)
*S. aureus* 15772	>67.3(>100)	7.57(18.75)	10.09(18.75)	0.28(1.17)
*S. aureus* 15192	0.40(0.59)	7.57(18.75)	0.63(1.17)	0.55(2.34)
VRSA 11	>67.3(>100)	15.14(37.5)	20.19(37.5)	2.21(9.38)
VRSA 52	>67.3(>100)	3.79(9.38)	5.05(9.38)	1.10(4.69)
*E. faecalis* ATCC29212	>67.3(>100)	30.28(75)	40.38(75)	4.42(18.75)
*E. faecalis* 2	>67.3(>100)	15.14(37.5)	20.19(37.5)	4.42(18.75)
*C. perfringens* ATCC13124	25.24(37.5)	7.57(18.75)	10.09(18.75)	2.21(9.38)
*B. cereus* CMCC63301	50.48(75)	7.57(18.75)	10.09(18.75)	2.21(9.38)
Gram‐negative				
*E. coli* ATCC25922	>67.3(>100)	1.89(4.69)	2.53(4.69)	0.55(2.34)
*E. coli* CMCC44102	>67.3(>100)	15.14(37.5)	20.19(37.5)	2.21(9.38)
*A. baumannii* ATCC19606	>67.3(>100)	3.79(9.38)	5.05(9.38)	0.28(1.17)
MDR *A. baumannii* 1	>67.3(>100)	3.79(9.38)	5.05(9.38)	1.10(4.69)
*K. pneumoniae* 9883	>67.3(>100)	15.14(37.5)	20.19(37.5)	4.42(18.75)
MDR *K. pneumoniae* 2	>67.3(>100)	7.57(18.75)	10.09(18.75)	4.42(18.75)
MDR *K. pneumoniae* 3	>67.3(>100)	3.79(9.38)	5.05(9.38)	2.21(9.38)
MDR *K. pneumoniae* 4	>67.3(>100)	7.57(18.75)	10.09(18.75)	4.42(18.75)
*P*. *aeruginosa* ATCC27853	>67.3(>100)	3.79(9.38)	5.05(9.38)	4.42(18.75)
*P. aeruginosa* CMCC10104	>67.3(>100)	7.57(18.75)	10.09(18.75)	4.42(18.75)
*S. typhimurium* ATCC14028	>67.3(>100)	7.57(18.75)	10.09(18.75)	4.42(18.75)
*S*. *typhimurium* CICC21483	>67.3(>100)	7.57(18.75)	10.09(18.75)	4.42(18.75)
*V. parahemolyticus* ATCC17802	50.48(75)	7.57(18.75)	10.09(18.75)	8.84(37.5)
*P*. *aeruginosa* 60357	>67.3(>100)	3.79(9.38)	5.05(9.38)	2.21(9.38)
*P*. *aeruginosa* 52097	>67.3(>100)	3.79(9.38)	5.05(9.38)	2.21(9.38)
MDR *P*. *aeruginosa* 5	>67.3(>100)	1.89(4.69)	2.53(4.69)	2.21(9.38)
*S. flexneri* ATCC12022	50.48(75)	3.79(9.38)	5.05(9.38)	1.10(4.69)
*S. flexneri* CMCC51571	>67.3(>100)	3.79(9.38)	5.05(9.38)	2.21(9.38)
GM	55.08(81.83)	7.67(18.99)	9.26(17.20)	2.60(11.03)

^a)^ Minimum inhibitory concentration;

^b)^ The physically mixed combination group of vancomycin and MSI‐78. The molar concentration of the mixture is calculated by dividing the mass concentration by the average molecular weight, which is determined by dividing the total mass by the total number of moles; GM: Geometric mean of the MICs across the 29 bacterial strains. For GM value calculation, MIC values exceeding 100 µg mL^−1^ were defaulted to 100 µg mL^−1^.

**Figure 2:**
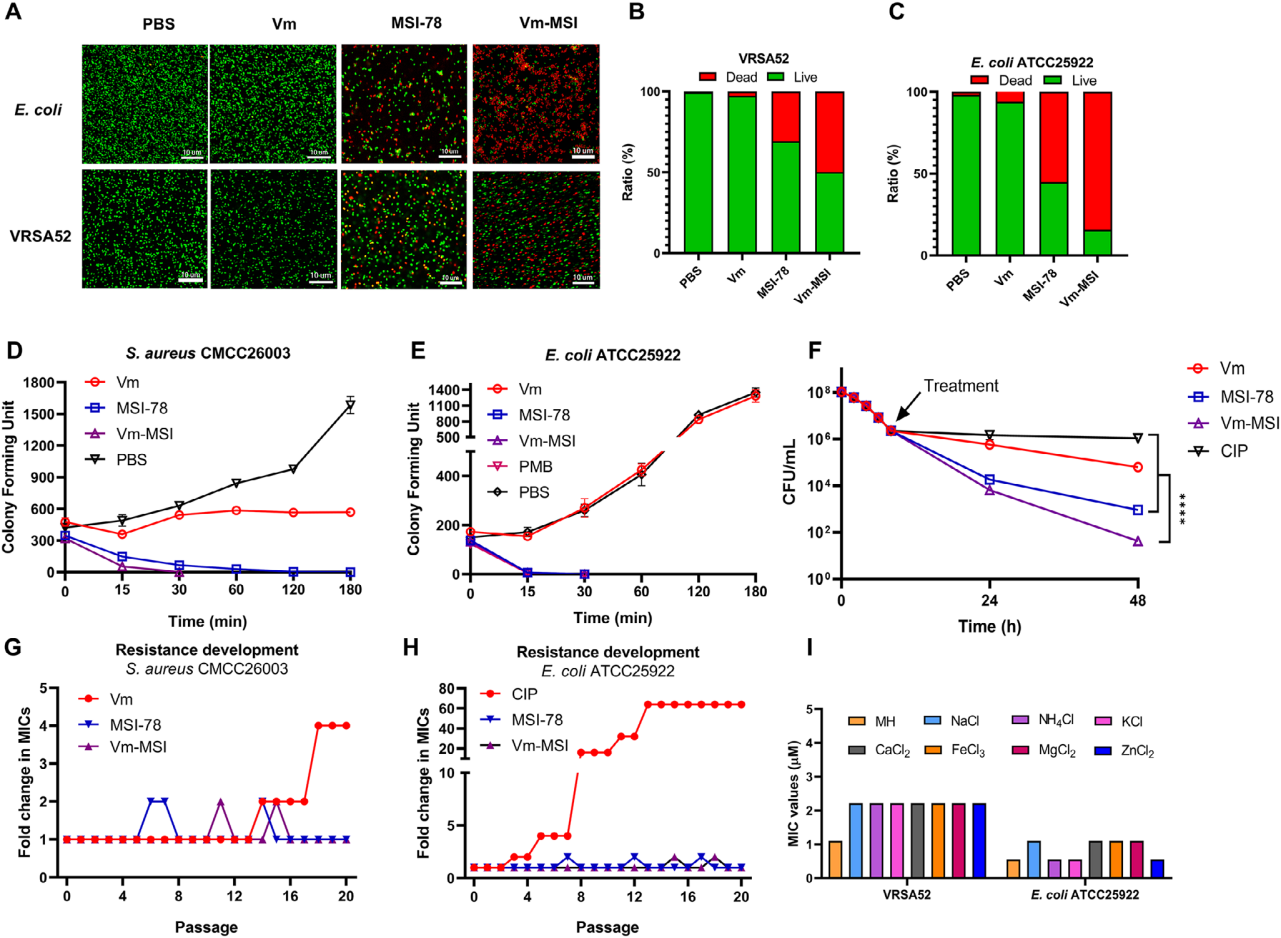
In vitro antimicrobial activity, persister killing activity, resistance development, and antimicrobial stability of Vm‐MSI. A) Live/dead staining of *E. coli* ATCC25922 and VRSA 52 was performed using PI/SYTO9. Bacteria were co‐incubated with vancomycin, MSI‐78, or Vm‐MSI at a final concentration of 4 µm for 1 h, followed by imaging with a laser confocal microscope. Green represents live bacteria, and red represents dead bacteria. Each result is representative of three independent experiments. B,C) Quantitative analysis of live/dead bacteria from the above experiments. D,E) Time‐kill kinetics of vancomycin, MSI‐78, and Vm‐MSI against *S. aureus* CMCC26003 and *E. coli* ATCC25922, using polymyxin B as a positive control, with a final concentration of 4 µm. Each value represents the median of three independent experiments. F) Antimicrobial activity of vancomycin, MSI‐78, and Vm‐MSI against persister cells. *S. aureus* CMCC26003 was incubated with 20× MIC ciprofloxacin for 8 h, followed by washing away residual ciprofloxacin and continued incubation with 4 µm of the respective drug for 48 h. Each value represents the median of three independent experiments. Statistical comparisons among multiple groups were performed using one‐way ANOVA followed by Tukey's post hoc test for pairwise comparisons.^****^ *p* < 0.0001. G,H) Resistance development in *S. aureus* CMCC26003 and *E. coli* ATCC25922 at sub‐MIC concentrations of vancomycin, MSI‐78, Vm‐MSI, or ciprofloxacin. I) MICs of Vm‐MSI in MH medium with physiological salt ion concentrations against VRSA 52 and *E. coli* ATCC25922. Each value represents the median of three independent experiments. n = 3 biological replicates for Figure 2D–F. The results are expressed as mean ± S.D. Source data are provided as a Source Data file.

To visualize the bactericidal effect of Vm‐MSI, we used the propidium iodide (PI)/SYTO9 staining method in combination with confocal laser scanning microscopy (CLSM) to assess bacterial growth after exposure to Vm‐MSI, following an established protocol (**Figure** **2**A–C).^[^ ^18^ ^]^ The nucleic acid dye PI emits red fluorescence upon binding to nucleic acids when bacterial cell membranes are disrupted, while the membrane dye SYTO9 emits green fluorescence by binding to intact membranes of viable cells. Bacterial viability was determined by quantifying the fluorescence intensity. Vm‐MSI demonstrated superior bactericidal efficacy compared to vancomycin and MSI‐78 alone. After incubating *E. coli* ATCC25922 with 4 µm Vm‐MSI for 1 h, over 80% of the bacteria were killed, while MSI‐78 and vancomycin achieved only 58% and 6% mortality, respectively. Similarly, treatment with 4 µm Vm‐MSI, MSI‐78, and vancomycin resulted in bacterial mortalities of 57%, 31%, and 2% for VRSA 52, respectively.

To further illustrate the superior bactericidal efficacy of Vm‐MSI, we performed a comparative analysis of the time‐kill kinetics between Vm‐MSI, vancomycin, and MSI‐78. For *S. aureus* CMCC26003, Vm‐MSI (4 µm) completely eradicated all viable bacteria within just 30 min, while MSI‐78 achieved the same result in 120 min. In contrast, vancomycin displayed significantly slower bactericidal activity and failed to eliminate the bacteria even after 180 min. For the Gram‐negative *E. coli* ATCC25922, Vm‐MSI and MSI‐78 demonstrated bactericidal efficiency comparable to that of polymyxin B, effectively eliminating all bacteria within 30 min. Vancomycin, however, showed no bactericidal activity, similar to the PBS‐treated group (Figure 2D,E; Figure S6, Supporting Information).

Persister cells represent a subpopulation of bacteria that have developed adaptive mechanisms to survive exposure to lethal antibiotics. The presence of these cells increases the risk of recurrent and relapsing infections, which, in turn, raises the likelihood of developing antibiotic resistance during treatment.^[^ ^31^ ^]^ In this study, we evaluated the antimicrobial activity of Vm‐MSI against persister cells. *S. aureus* CMCC26003 was exposed to 20× MIC ciprofloxacin to induce the formation of persister cells, and the efficacy of Vm‐MSI in eradicating these cells was assessed and compared to vancomycin and MSI‐78 alone.^[^ ^18^ ^]^ Vancomycin exhibited limited bactericidal activity, leading to only a one‐order reduction in the number of persister cells after 48 h of incubation. In contrast, MSI‐78 showed greater efficacy, achieving a three‐order reduction in persister cell numbers. Notably, Vm‐MSI demonstrated a significantly stronger bactericidal effect, reducing the number of persister cells by more than four orders of magnitude (Figure 2F).

Prolonged exposure of bacteria to sub‐MIC concentrations of antibiotics can readily induce the development of resistance, thereby highlighting the significance of resistance propensity as a critical criterion in evaluating antimicrobial drugs. In this study, we compared the propensity of bacteria to develop resistance to Vm‐MSI versus MSI‐78, vancomycin, and conventional antibiotics. *S. aureus* CMCC26003 and *E. coli* ATCC25922 were cultured at sub‐MIC concentrations of these drugs, and their MIC values were monitored over 20 generations. The MIC values of Vm‐MSI and MSI‐78 against both *S. aureus* and *E. coli* remained stable across 20 generations, whereas vancomycin exhibited a four‐fold increase in MIC against *S. aureus*, and ciprofloxacin showed a 64‐fold increase in MIC against *E. coli* (Figure 2G,H). These findings suggest that Vm‐MSI offers a significant advantage over conventional antibiotics in mitigating the development of bacterial resistance.

Previous studies have shown that the antimicrobial activity of some cationic AMPs is hindered by metal ions, as the electrostatic repulsion between the ions and the AMPs' similar charges interferes with their function.^[^ ^32^ ^]^ To evaluate the potential interference of metal salt ions on the antimicrobial activity of Vm‐MSI, we selected seven common cations present in the human body and examined their effect on Vm‐MSI's efficacy. The results indicated that these cations had minimal impact on the antimicrobial activity of Vm‐MSI against VRSA 52 and *E. coli* ATCC25922 (Figure 2I). The MIC values remained relatively stable, with only a slight increase or decrease of up to two‐fold. These findings suggest that Vm‐MSI's antimicrobial activity is not significantly affected by physiological salt ions, making it a promising candidate for the treatment of in vivo infections.

**Figure 3:**
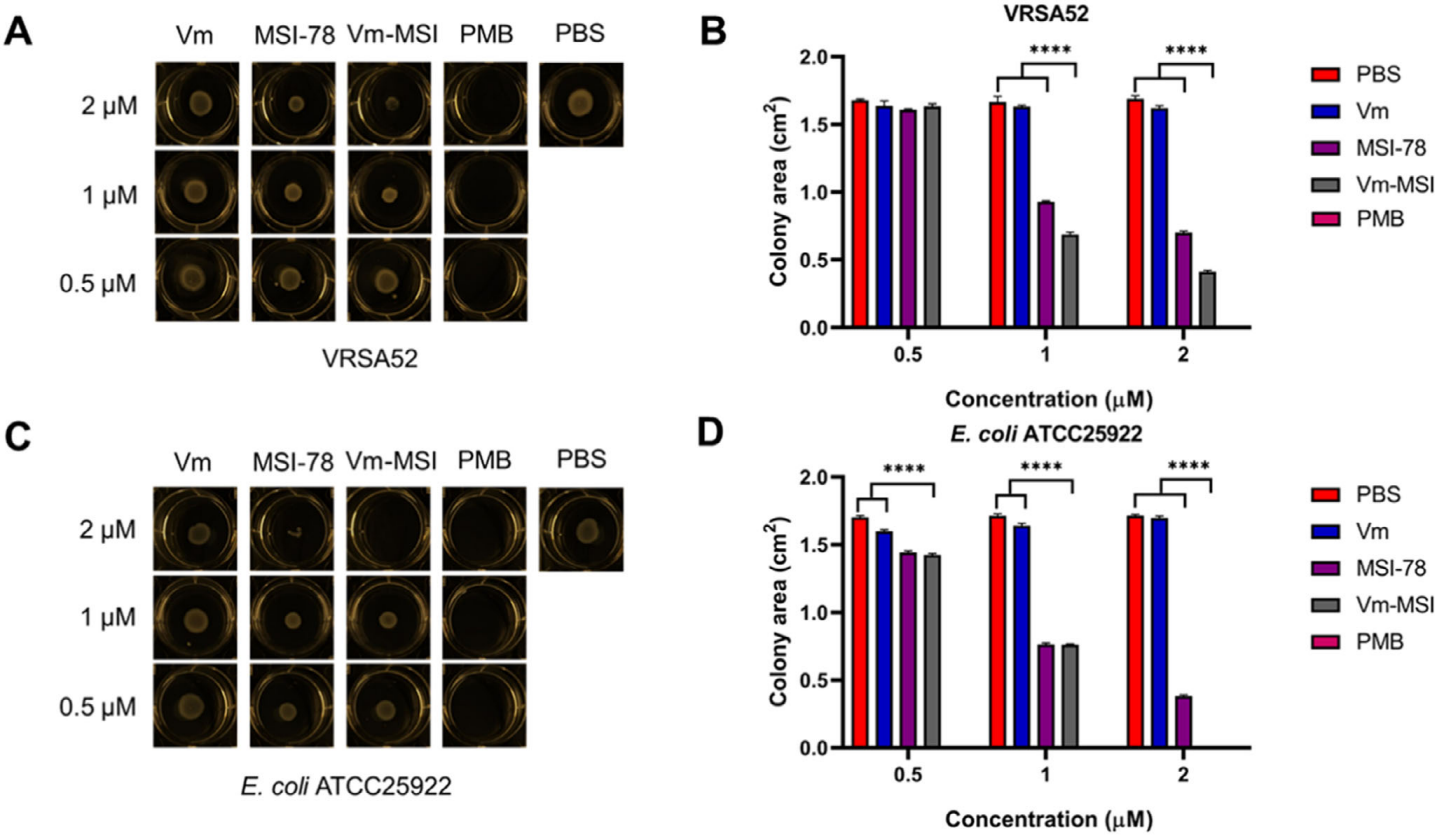
Vm‐MSI inhibits bacterial swimming motility. A,C) Colony morphology formed by bacterial swimming motion on solid medium containing vancomycin, MSI‐78, Vm‐MSI, and polymyxin B (positive control) at final concentrations of 0.5 µm, 1 µm, and 2 µm. A 5 µL drop containing 1 × 10⁶ CFU mL^−1^ of VRSA 52 and *E. coli* ATCC25922 was placed on the medium and incubated for 16 h at 37 °C. The size of the resulting colonies was measured. Each result represents one of three independent experiments. B,D) Quantification of the colony areas for each treatment group is shown in Figures A and C (n = 3 biological replicates). Statistical comparisons among multiple groups were performed using one‐way ANOVA followed by Tukey's post hoc test for pairwise comparisons. ^****^ *p* < 0.0001. The results are expressed as mean ± S.D. Source data are provided as a Source Data file.

To survive in complex environments, bacteria have evolved unconventional motor systems. Swimming motility, driven by flagella, allows bacteria to move from the inoculation site to surrounding areas on the medium's surface. This movement is essential for bacterial survival, growth, virulence, biofilm formation, and intra‐ and interspecies interactions.^[^ ^33^, ^34^ ^]^ We conducted an analysis to assess the effect of Vm‐MSI on bacterial swimming motility at sub‐MIC concentrations. Results showed that Vm‐MSI inhibits bacterial swimming motility in a concentration‐dependent manner, as evidenced by the significant reduction in colony area formed by bacterial movement (**Figure** **3**A–D). Notably, the colony area in the Vm‐MSI treatment group was significantly smaller compared to both the MSI‐78 and vancomycin treatment groups at equivalent concentrations. As to the positive control polymyxin B group, because the drug concentrations used in the assay were beyond lethal concentrations toward the bacteria, no discernible colony morphology was observed. This finding suggests that Vm‐MSI more effectively inhibits bacterial swimming motility than vancomycin or MSI‐78 alone.

### 2.3 Anti‐Biofilm Activity of Vm‐MSI

**Figure 4:**
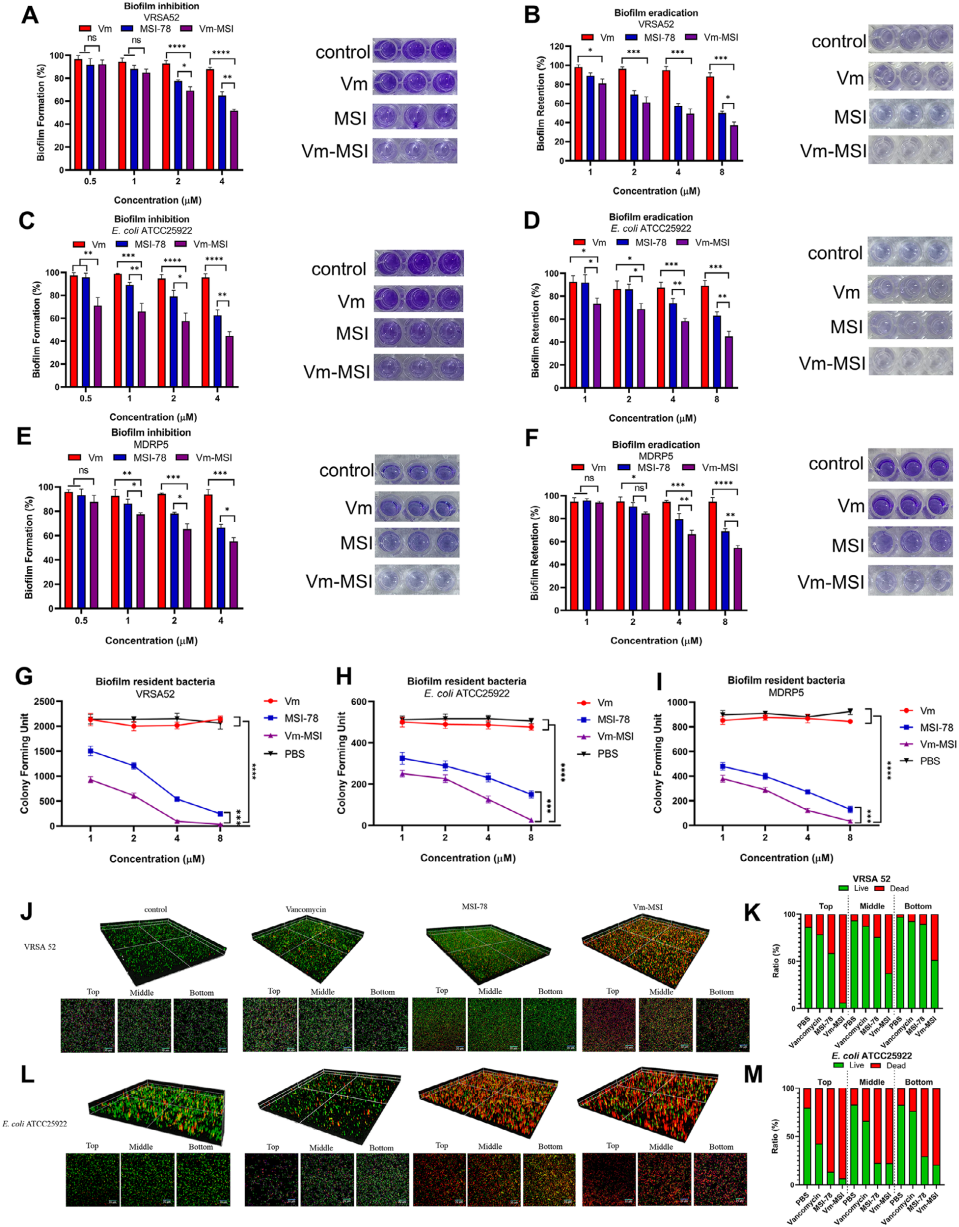
Anti‐biofilm activity of Vm‐MSI. A, C, E) Biofilm inhibition activity of vancomycin, MSI‐78, and Vm‐MSI against VRSA 52, *E. coli* ATCC25922, and MDR *P. aeruginosa* 5. B, D, F) Biofilm eradication activity of vancomycin, MSI‐78, and Vm‐MSI against the same strains. In the images, a darker blue‐violet color indicates more residual biofilm. G, H, I) Antimicrobial activity of vancomycin, MSI‐78, and Vm‐MSI against biofilm‐resident bacteria of VRSA 52, *E. coli* ATCC25922, and MDR *P. aeruginosa* 5. J) Z‐stack confocal images and depth‐specific representative views of VRSA 52 biofilms following treatment with vancomycin, MSI‐78, or Vm‐MSI (8 µm). K) Quantitative analysis of live/dead cell ratios at the surface, middle, and bottom layers of VRSA 52 biofilms. L) Z‐stack confocal images and depth‐specific representative views of *E. coli* ATCC25922 biofilms following treatment with vancomycin, MSI‐78, or Vm‐MSI (8 µm). (M) Quantitative analysis of live/dead cell ratios at the surface, middle, and bottom layers of *E. coli* ATCC25922 biofilms. Each value represents the median of three independent experiments. Statistical comparisons among multiple groups were performed using one‐way ANOVA followed by Tukey's post hoc test for pairwise comparisons. ^*^ *p* < 0.1, ^**^ *p* < 0.01, ^***^ *p* < 0.001, ^****^ *p* < 0.0001. n = 3 biological replicates for Figure 4A–I. The results are expressed as mean ± S.D. Source data are provided as a Source Data file.

Biofilm is a bacterial‐produced polymer composed of polysaccharides, proteins, DNA, and other biomolecules.^[^ ^20^ ^]^ Biofilm presence obstructs the interaction and targeting of antimicrobial agents, promoting drug resistance and the persistence of chronic infections.^[^ ^20^, ^35^ ^]^ We assessed the anti‐biofilm activity of Vm‐MSI against three bacterial strains: VRSA 52, *E. coli* ATCC25922, and MDR *P. aeruginosa* 5 using crystalline violet biofilm inhibition and eradication assays. Vancomycin displayed no noticeable anti‐biofilm activity (**Figure** **4**A–F). In contrast, both Vm‐MSI and MSI‐78 demonstrated concentration‐dependent inhibition of biofilm formation, with Vm‐MSI showing superior efficacy. At 4 µm, Vm‐MSI significantly reduced biofilm formation in VRSA, *E. coli*, and MDR *P. aeruginosa* by 60%, 60%, and 50%, respectively, compared to MSI‐78′s inhibition rates of 40%, 50%, and 40% (Figure 4A,C,E). Consistently, Vm‐MSI was also highly effective in eradicating preformed biofilms. At 8 µm, Vm‐MSI cleared 70% of VRSA biofilms, 60% of *E. coli*, and 50% of MDR *P. aeruginosa* biofilms (Figure 4B,D,F).

To further validate the anti‐biofilm activity of the conjugate across different bacterial backgrounds, we selected six additional strains based on their varying susceptibility to vancomycin and MSI‐78. Remarkably, Vm‐MSI displayed the strongest inhibition and eradication effects regardless of resistance profile. For instance, Vm‐MSI was highly effective not only against MRSA ATCC43300, which shows relatively weak resistance to vancomycin (Figure S7A, B, Supporting Information), but also against *E. faecalis* ATCC29212, which exhibits stronger resistance to MSI‐78 (Figure S7C, D, Supporting Information). In addition, we confirmed that Vm‐MSI retained superior activity against other representative strains, including *S. aureus* 15772 (Figure S7E, F, Supporting Information), *P. aeruginosa* ATCC27853 (Figure S7G, H, Supporting Information), *A. baumannii* ATCC19606 (Figure S7I, J, Supporting Information), and *K. pneumoniae* 9883 (Figure S7K, L, Supporting Information). Collectively, these findings demonstrate that Vm‐MSI consistently achieves the most potent biofilm inhibition and eradication across diverse Gram‐positive and Gram‐negative pathogens.

Furthermore, to thoroughly evaluate the anti‐biofilm activity of Vm‐MSI, we employed the triphenyl tetrazolium chloride (TTC) assay to assess both biofilm inhibition and eradication.^[^ ^36^ ^]^ The colorless TTC reagent reacts with bacterial biofilms to form a reddish‐brown complex visible to the naked eye (Figure S8, Supporting Information). Consistent with the results obtained from crystalline violet assays, Vm‐MSI and MSI‐78 exhibited concentration‐dependent inhibitory and eradication effects on biofilms, with Vm‐MSI demonstrating superior efficacy. In the Vm‐MSI treatment group, the wells remained almost transparent, while the MSI‐78 group showed a light red color, and the vancomycin group appeared reddish‐brown (Figure S8, Supporting Information). These TTC assay results support the previous findings, confirming that Vm‐MSI demonstrates superior anti‐biofilm activity compared to MSI‐78 and vancomycin alone.

At wound sites of catheter‐associated urinary tract infections and on the surfaces of hospital surgical instruments, many otherwise antibiotic‐sensitive pathogenic bacteria develop resistance due to the protective shelter of biofilms. These bacteria are referred to as biofilm‐resident bacteria.^[^ ^20^ ^]^ This phenomenon presents a major challenge in the management of clinical infections.^[^ ^37^ ^]^ We evaluated the antimicrobial efficacy of Vm‐MSI against biofilm‐resident bacteria. Vm‐MSI and MSI‐78 demonstrated concentration‐dependent antimicrobial activity against biofilm‐resident bacteria, including VRSA, *E. coli*, and MDR *P. aeruginosa*, with Vm‐MSI demonstrating superior efficacy (Figure 4G,H,I). Vm‐MSI eradicated over 50% of biofilm‐resident bacteria at a concentration of 1 µm, and at 8 µm, it successfully eliminated 99% of the biofilm‐resident bacteria. In contrast, the vancomycin‐treated group showed no significant biofilm‐resident bactericidal effect and performed similarly to the PBS control.

To visualize the bactericidal effects of different treatments on biofilm‐resident bacteria, we employed CLSM with SYTO9/PI staining. We further examined the impact of vancomycin, MSI‐78, and Vm‐MSI (8 µm) on biofilms formed under static conditions, focusing on bacterial viability across different depths. The results, as shown in Figure 4J–M, revealed distinct differences in penetration and bactericidal activity among the treatments. For VRSA 52, vancomycin showed minimal activity, with killing largely restricted to the surface layer (25% dead cells), closely resembling the control group. MSI‐78 displayed improved efficacy, effectively clearing surface bacteria and achieving 25% killing in the middle layer, but little effect in deeper layers. In contrast, Vm‐MSI penetrated throughout the biofilm and exerted strong bactericidal activity, eliminating 95% of surface bacteria, 60% of middle‐layer bacteria, and 50% of the bottom‐layer bacteria (Figure 4J,K).

A similar trend was observed for *E. coli* ATCC25922. Vancomycin exhibited limited efficacy, with 60% killing at the surface but rapidly declining activity in deeper layers, with only 20% killing at the bottom, nearly identical to the control. Both MSI‐78 and Vm‐MSI achieved near‐complete clearance of surface bacteria and maintained substantial bactericidal effects in deeper regions, with Vm‐MSI killing more than 75% of the bottom‐layer bacteria (Figure 4L,M). Collectively, these results confirm that Vm‐MSI not only retains but further enhances the anti‐biofilm capacity of MSI‐78, effectively penetrating biofilms and killing bacteria across all depths.

### 2.4 Synergistic Antimicrobial Activity of Vm‐MSI with Conventional Antibiotics

**Figure 5:**
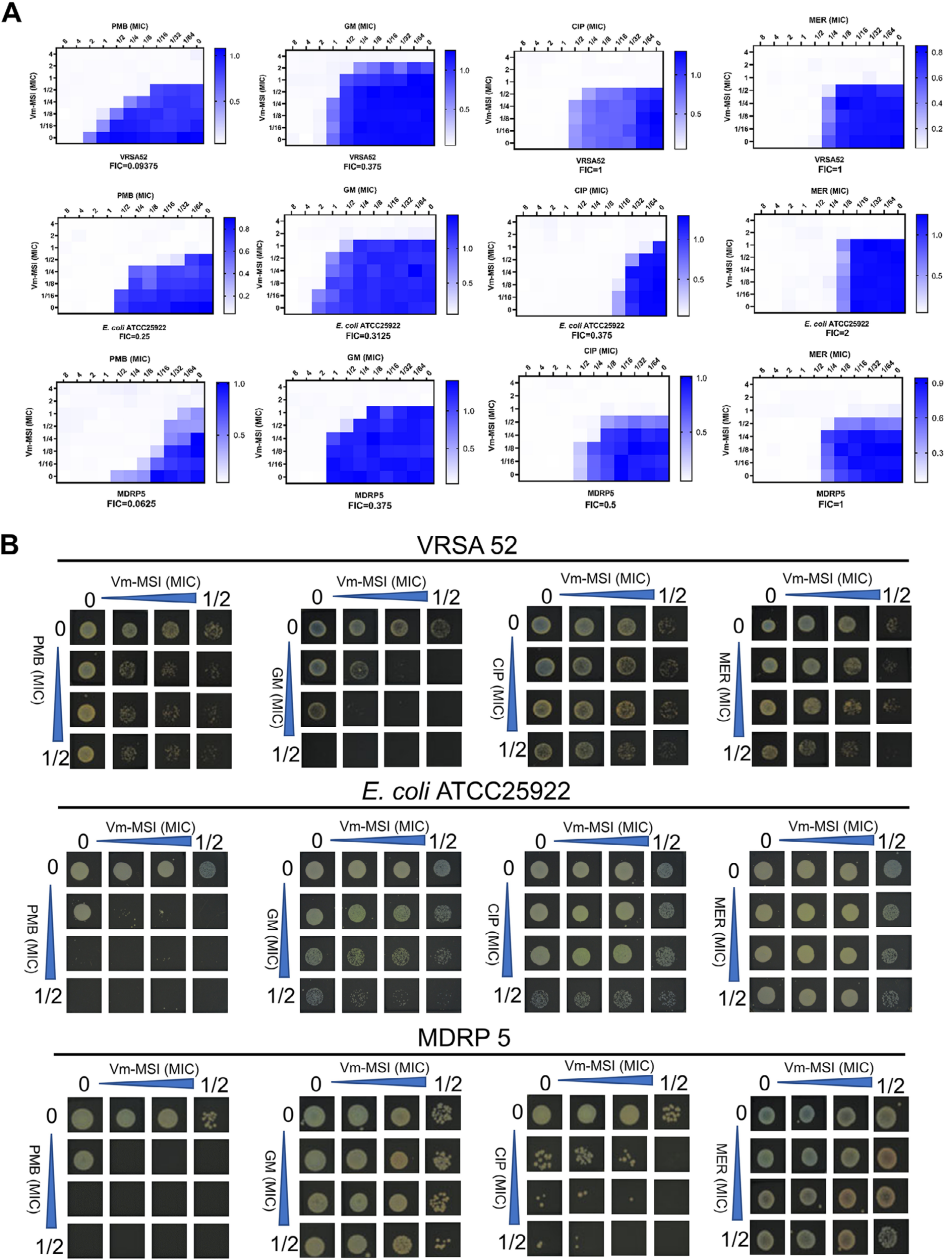
Synergistic antimicrobial effect between Vm‐MSI and conventional antibiotics with different mechanisms. A) The synergistic effects of Vm‐MSI with polymyxin B, ciprofloxacin, gentamicin, and meropenem were evaluated using the checkerboard method. The heat map represents the bacterial growth index (OD_600_) in the corresponding wells, with varying color shades indicating the extent of bacterial growth. Three bacterial strains were tested: VRSA 52, *E. coli* ATCC25922, and MDR *P. aeruginosa* 5. B) The synergistic activity of Vm‐MSI with the antibiotics was further validated using the spot titer assay. Following co‐administration of Vm‐MSI and the four antibiotics, bacterial cultures were incubated for 3 h, and 5 µL of the bacterial solution was subsequently transferred to fresh LB medium for colony morphology observation. From top to bottom, the bacterial strains tested were VRSA 52, *E. coli* ATCC25922, and MDR *P. aeruginosa* 5. PMB: polymyxin B; GM: gentamicin; CIP: ciprofloxacin; MER: meropenem. Each value represents the median of three independent experiments. Source data are provided as a Source Data file.

AMPs have shown promising synergistic effects when combined with conventional antibiotics. This combination therapy presents an effective strategy for combating multidrug‐resistant bacterial infections.^[^ ^12^, ^38^, ^39^ ^]^ We assessed the synergistic antimicrobial effect of Vm‐MSI in combination with various representative antibiotics. Conventional antibiotics act through distinct mechanisms; for instance, meropenem inhibits cell wall synthesis, polymyxin B disrupts cell membranes, ciprofloxacin blocks DNA synthesis, and gentamicin impairs protein synthesis. In this study, we combined Vm‐MSI with these antibiotics to evaluate their combined antimicrobial effects against three bacterial strains, of which two are drug‐resistant strains. The checkerboard method was used to analyze the synergy between Vm‐MSI and these four antibiotics. Synergy was quantified using the fractional inhibitory concentration (FIC) index: FIC ≤ 0.5 indicates synergy, FIC > 2 indicates antagonism, 0.5 < FIC ≤ 1 indicates additivity, and 1 < FIC ≤ 2 suggests no interaction.^[^ ^12^ ^]^ The combination of Vm‐MSI with polymyxin B, ciprofloxacin, and gentamicin demonstrated either synergistic or additive antimicrobial effects, while no synergistic interaction was observed with meropenem (**Figure** **5**A). Notably, the combination with polymyxin B exhibited the strongest synergy, with a 16 fold increase in activity against VRSA 52 when co‐administered with Vm‐MSI. Similarly, this combination resulted in an 8 fold enhancement against *E. coli* ATCC25922 and an impressive 32 fold improvement against MDR *P. aeruginosa* 5 (Figure 5A). To further illustrate the synergistic effect, the spot titer assay was performed, and the results confirmed that the antimicrobial activity in the combination groups was significantly greater than that of the individual antibiotics (Figure 5B). In summary, these findings reveal that Vm‐MSI not only possesses strong standalone antimicrobial efficacy but also enhances the activity of many conventional antibiotics through synergistic interactions.

### 2.5 Multifaceted Antimicrobial Mechanisms of Vm‐MSI

**Figure 6:**
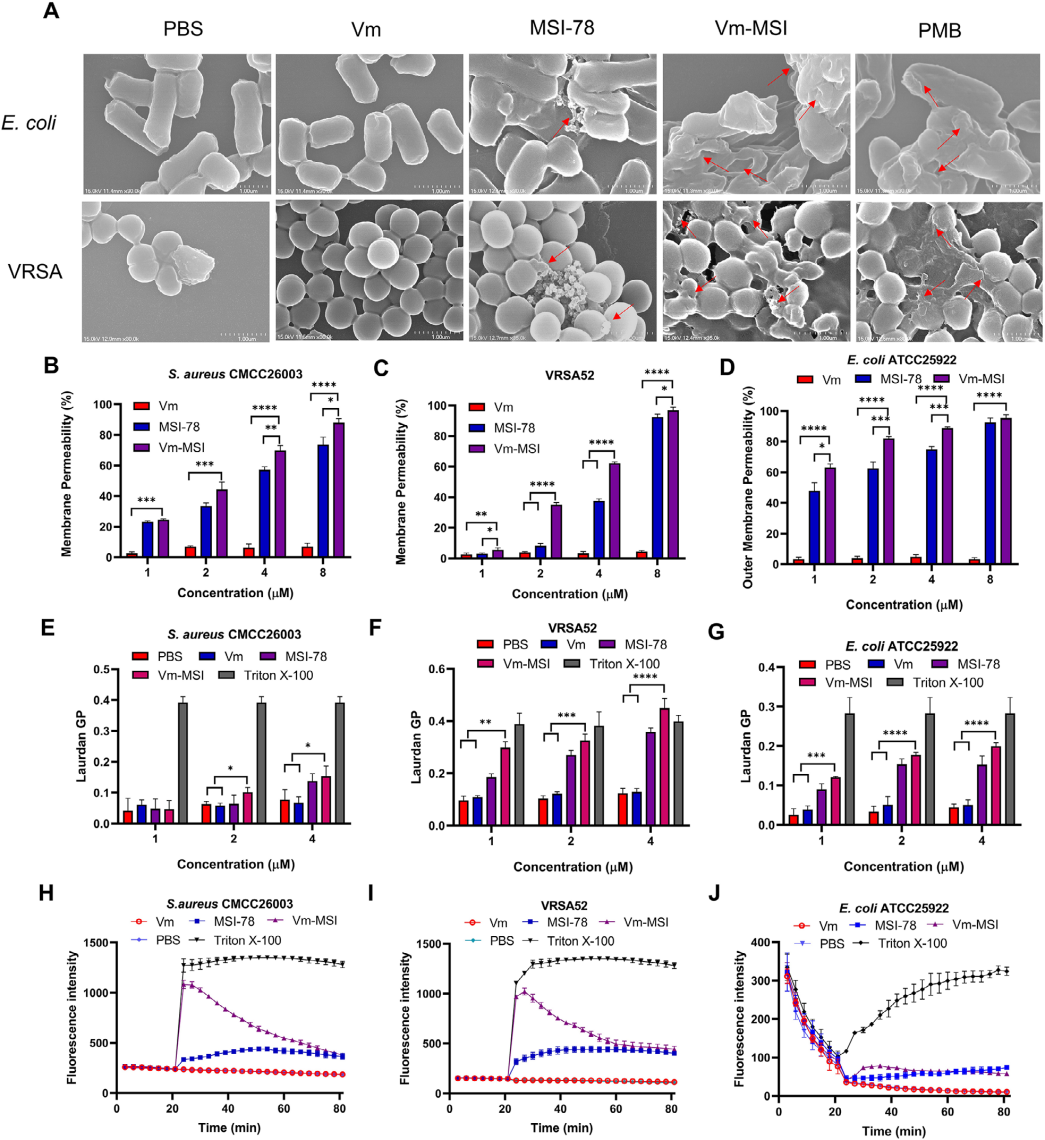
Vm‐MSI disrupts bacterial cell membranes. A) Scanning electron microscopy (SEM) was utilized to visualize *E. coli* ATCC25922 and VRSA 52 cells after one‐h exposure to 4 µm vancomycin, MSI‐78, and Vm‐MSI. The images reveal areas of cell membrane damage, indicated by red arrows. B–D) The impact of vancomycin, MSI‐78, and Vm‐MSI on the cell membrane permeability of *S. aureus* CMCC26003, VRSA 52, and *E. coli* ATCC25922 was assessed. For *S. aureus* CMCC26003 and VRSA 52, propidium iodide was employed as a fluorescence dye for the detection of cytomembrane permeability, with an excitation wavelength of 535 nm and an emission wavelength of 615 nm. In contrast, N‐phenyl‐1‐naphthylamine was used for the detection of outer membrane permeability of *E. coli* ATCC25922, with an excitation wavelength of 350 nm and an emission wavelength of 420 nm. E–G) The effects of vancomycin, MSI‐78, and Vm‐MSI on the fluidity of the cell membranes of *S. aureus* CMCC26003, VRSA 52, and *E. coli* ATCC25922 were examined. Membrane fluidity changes were detected using a Laurdan probe, with an excitation wavelength of 350 nm and emission wavelengths of 435 and 490 nm. H–J) The influence of vancomycin, MSI‐78, and Vm‐MSI (4 µm) on the membrane potential of *S. aureus* CMCC26003, VRSA 52, and *E. coli* ATCC25922 was investigated. Bacterial membrane depolarization was measured using DiSC_3_(5), with an excitation wavelength of 622 nm and an emission wavelength of 670 nm. Each value represents the median of three independent experiments. Statistical comparisons among multiple groups were performed using one‐way ANOVA followed by Tukey's post hoc test for pairwise comparisons, with ^*^ *p* < 0.05, ^**^ *p* < 0.01, ^***^ *p* < 0.001, and ^****^ *p* < 0.0001. n = 3 biological replicates for Figure 6B–J. The results are expressed as mean ± S.D. Source data are provided as a Source Data file.

To investigate the underlying mechanisms of Vm‐MSI's antibacterial effects, we first utilized scanning electron microscopy (SEM) to observe bacterial morphology post‐treatment. Significant differences were noted in the cell membrane structure of VRSA and *E. coli* treated with Vm‐MSI or MSI‐78, resembling the effects observed with the positive control, polymyxin B. In contrast, the bacterial membranes remained intact in the vancomycin and PBS control groups. Treatment with Vm‐MSI or MSI‐78 caused significant membrane disruption, as evidenced by pronounced structural damage observed under SEM (**Figure** **6**A). The images revealed that Vm‐MSI induces extensive membrane deformation and lysis, leading to bacterial death. This effect appears to be derived from its parent peptide MSI‐78, but with distinguishable morphological differences. Compared to MSI‐78, Vm‐MSI treatment resulted in more severe membrane collapse and surface disintegration, suggesting enhanced disruptive capability. To further explore the mechanism by which Vm‐MSI compromises membrane integrity, we assessed changes in bacterial cell membrane permeability after drug treatment using PI and NPN fluorescent probes. The results showed that vancomycin did not affect membrane permeability, whereas Vm‐MSI and MSI‐78 caused a concentration‐dependent increase in permeability. Notably, Vm‐MSI exhibited a stronger capacity to increase membrane permeability than MSI‐78 at equivalent concentrations (Figure 6B–D). Previous studies have identified alterations in cell membrane fluidity as a key indicator of membrane disruption, with Laurdan GP serving as a marker for these changes.^[^ ^40^, ^41^ ^]^ We investigated the changes in bacterial cell membrane fluidity following treatment with Vm‐MSI. The findings indicated that vancomycin had no effect on membrane fluidity, while both MSI‐78 and Vm‐MSI significantly reduced membrane fluidity in a concentration‐dependent manner, as demonstrated by an increase in Laurdan GP values (Figure 6 E–G). Additionally, disruption of the cell membrane caused notable alterations in membrane potential. This depolarization plays a critical role in both the structural and functional impairment of bacterial cell membranes, contributing to the antimicrobial activity of AMPs.^[^ ^42^ ^]^ DiSC_3_(5) is a membrane‐bound fluorescent probe that is highly sensitive to even minor changes in bacterial membrane potential or structural conformation.^[^ ^43^ ^]^ Thus, we examined the effect of Vm‐MSI on bacterial membrane potential using the DiSC_3_(5) probe. Both Vm‐MSI and MSI‐78 induced significant depolarization of the cell membranes in *S. aureus* CMCC26003 and VRSA, compared to the vancomycin‐treated and untreated control groups (Figure 6H–J). Notably, the membrane depolarization caused by Vm‐MSI was more pronounced than that of MSI‐78 at equivalent concentrations.

**Figure 7:**
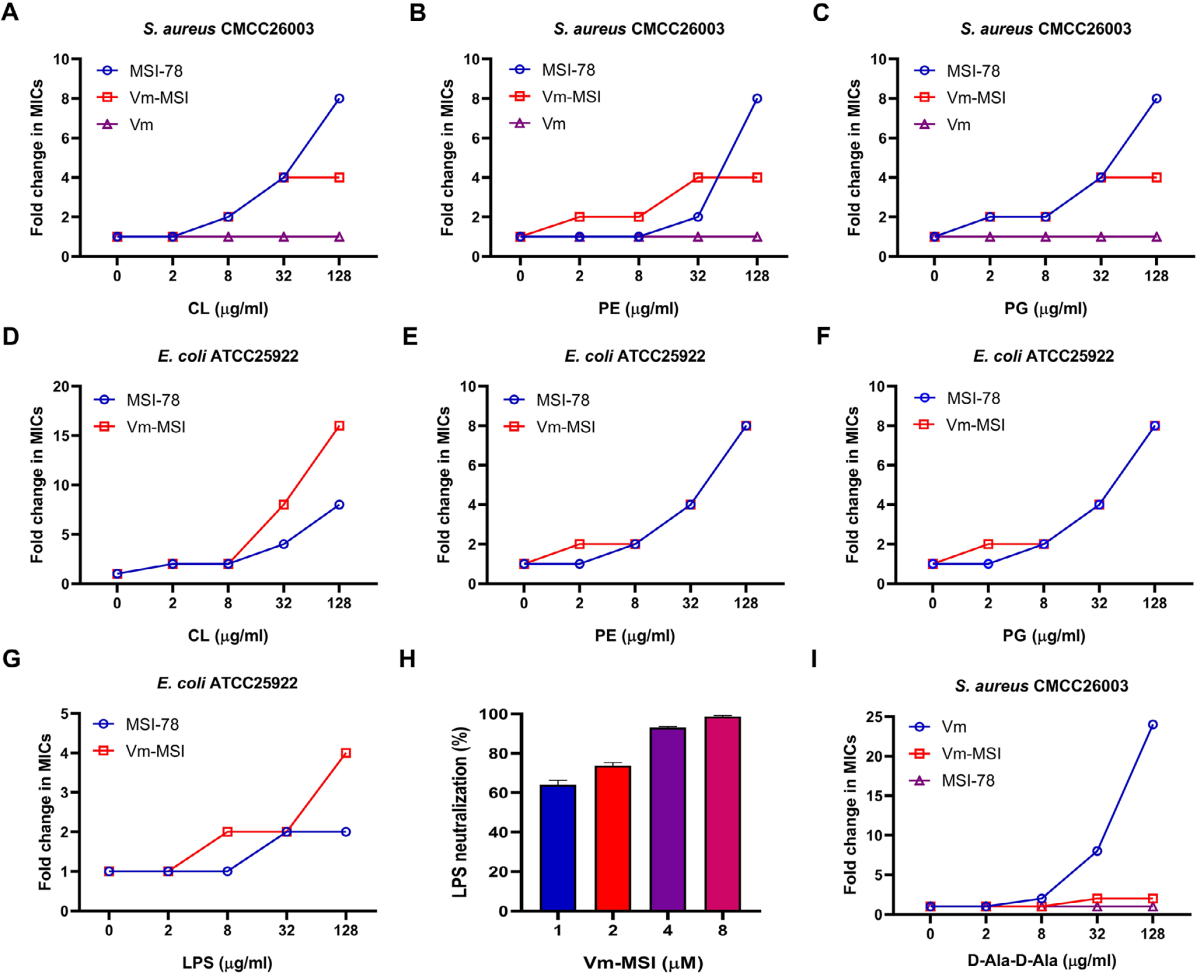
Vm‐MSI combined the dual antimicrobial mechanisms of vancomycin and MSI‐78. A–G) Antimicrobial blocking assay. Changes in the MICs of Vm‐MSI, MSI‐78, and vancomycin against *S. aureus* CMCC26003 and *E. coli* ATCC25922 were assessed in the presence of varying concentrations of cardiolipin (CL), phosphatidylethanolamine (PE), phosphoglyceride (PG), and lipopolysaccharide (LPS) ranging from 0 to 128 µg mL^−1^. H) LPS‐neutralizing assay conducted using the LAL colorimetric kit. I) Changes in the MICs of Vm‐MSI, MSI‐78, and vancomycin against *S. aureus* CMCC26003 were evaluated in the presence of different concentrations of D‐Ala‐D‐Ala (0 to 128 µg mL^−1^). Each value represents the median of three independent experiments. n = 3 biological replicates for Figure 7H. The results are expressed as mean ± S.D. Source data are provided as a Source Data file.

The results indicate that Vm‐MSI exerts bactericidal effects partly by disrupting bacterial cell membranes. It has been proven that the positive charge of AMPs facilitates their binding to the negatively charged components of the bacterial cell membrane.^[^ ^42^ ^]^ The bacterial cell membrane consists of a complex mixture of phospholipids, proteins, and carbohydrates. The negatively charged components predominantly include phosphatidylethanolamine (PE), phosphoglyceride (PG), cardiolipin (CL), and lipopolysaccharide (LPS).^[^ ^44^ ^]^ To determine the specific membrane components targeted by Vm‐MSI, we performed an antimicrobial blocking assay using the four aforementioned components. The addition of PE, PG, CL, and LPS significantly reduced the antimicrobial activity of Vm‐MSI in a concentration‐dependent manner (**Figure** **7**A–G). This conclusion was further validated through LPS neutralization experiments, which demonstrated that Vm‐MSI achieved an impressive LPS neutralization rate exceeding 60% at a concentration of 1 µm (Figure 7H). These results suggest that Vm‐MSI disrupts the cell membrane by first binding to the negatively charged components.

Additionally, we conducted an antimicrobial blockade experiment using D‐Ala‐D‐Ala. As shown in Figure 7I, the exogenous addition of D‐Ala‐D‐Ala resulted in a more than 2 fold increase in the MIC value of Vm‐MSI and over a 25 fold increase in the MIC value of vancomycin, while exerting no effect on the MIC value of MSI‐78. Since D‐Ala‐D‐Ala is the target of vancomycin, the exogenous addition of D‐Ala‐D‐Ala competitively inhibited the antimicrobial activity of Vm‐MSI. This finding indicates that Vm‐MSI retains the antimicrobial mechanism of vancomycin.

**Figure 8:**
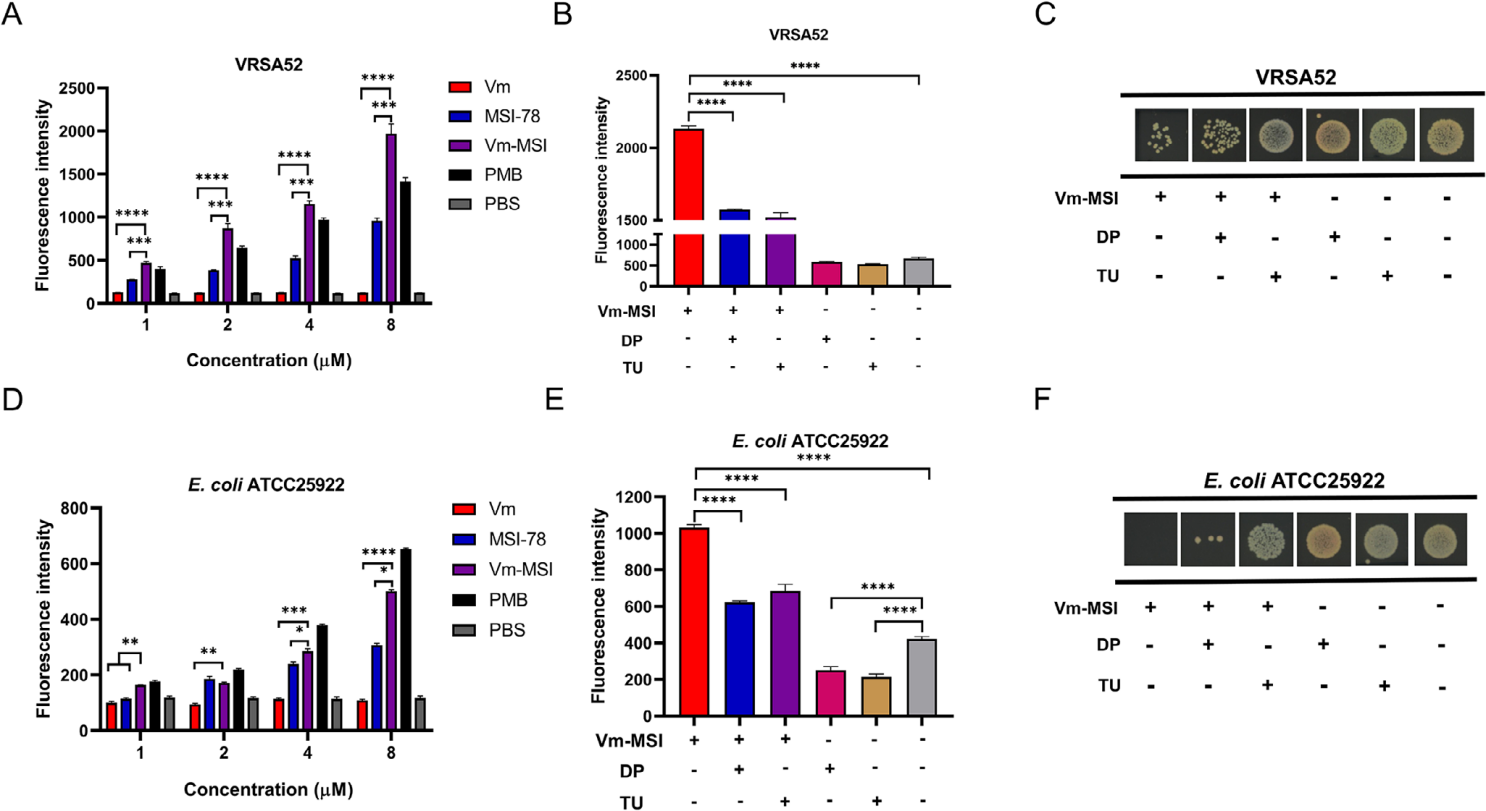
Vm‐MSI kills bacteria by promoting ROS production and bacterial oxidative damage. A, D) Bacterial intracellular ROS levels were detected using the DCFH‐DA fluorescent probe. Serial concentrations of Vm‐MSI were co‐incubated with VRSA 52 and *E. coli* ATCC25922, and the ROS production in bacteria was measured using this probe. PBS served as the negative control, and polymyxin B was used as the positive control. The DCFH‐DA probe was employed at a final concentration of 10 µm, with an excitation wavelength of 488 nm and an emission wavelength of 525 nm. B, C, E, F) The relationship between ROS levels and the bactericidal activity of Vm‐MSI was examined. In panels (B) and (E), ROS scavengers 2,2′‐bipyridine (DP) (300 mm) and thiourea (TU) (80 µm) were added prior to measuring ROS production in bacteria stimulated by Vm‐MSI (8 µm). In panels (C) and (F), the same scavengers were pre‐added before introducing Vm‐MSI (4 µm), followed by a one‐h co‐incubation, after which bacterial growth was observed. Statistical comparisons among multiple groups were performed using one‐way ANOVA followed by Tukey's post hoc test for pairwise comparisons, with ^*^ *p* < 0.05, ^**^ *p* < 0.01, ^***^ *p* < 0.001, and ^****^ *p* < 0.0001. Each value represents the median of three independent experiments. n = 3 biological replicates for Figure 8A, B, D, E. The results are expressed as mean ± S.D. Source data are provided as a Source Data file.

Reactive oxygen species (ROS) are byproducts produced during the normal metabolic processes of living organisms. However, the accumulation of ROS leads to oxidative stress, which in turn damages DNA, proteins, and other biomolecules.^[^ ^45^ ^]^ The bactericidal effects of some antibiotics and AMPs are reported to be mediated by the induction of intracellular ROS production in bacteria.^[^ ^46^, ^47^ ^]^ Thus, in the present study, ROS generation was evaluated during the antimicrobial action of Vm‐MSI. The results showed that while vancomycin did not induce ROS production, both Vm‐MSI and MSI‐78 significantly increased ROS levels in a dose‐dependent manner. Notably, Vm‐MSI induced substantially higher ROS levels than MSI‐78 at equivalent concentrations (**Figure** **8**A,D). To explore the relationship between ROS production and the antimicrobial activity of Vm‐MSI, ROS scavengers 2,2′‐bipyridine (DP) and thiourea (Tu) were incorporated in the antimicrobial assay. The addition of DP and Tu significantly reduced the bactericidal activity of Vm‐MSI (Figure 8B,C,E,F), indicating that ROS generation and accumulation play a key role in the bactericidal action of Vm‐MSI.

### 2.6 Transcriptomic and Targeted qPCR Analysis

**Figure 9:**
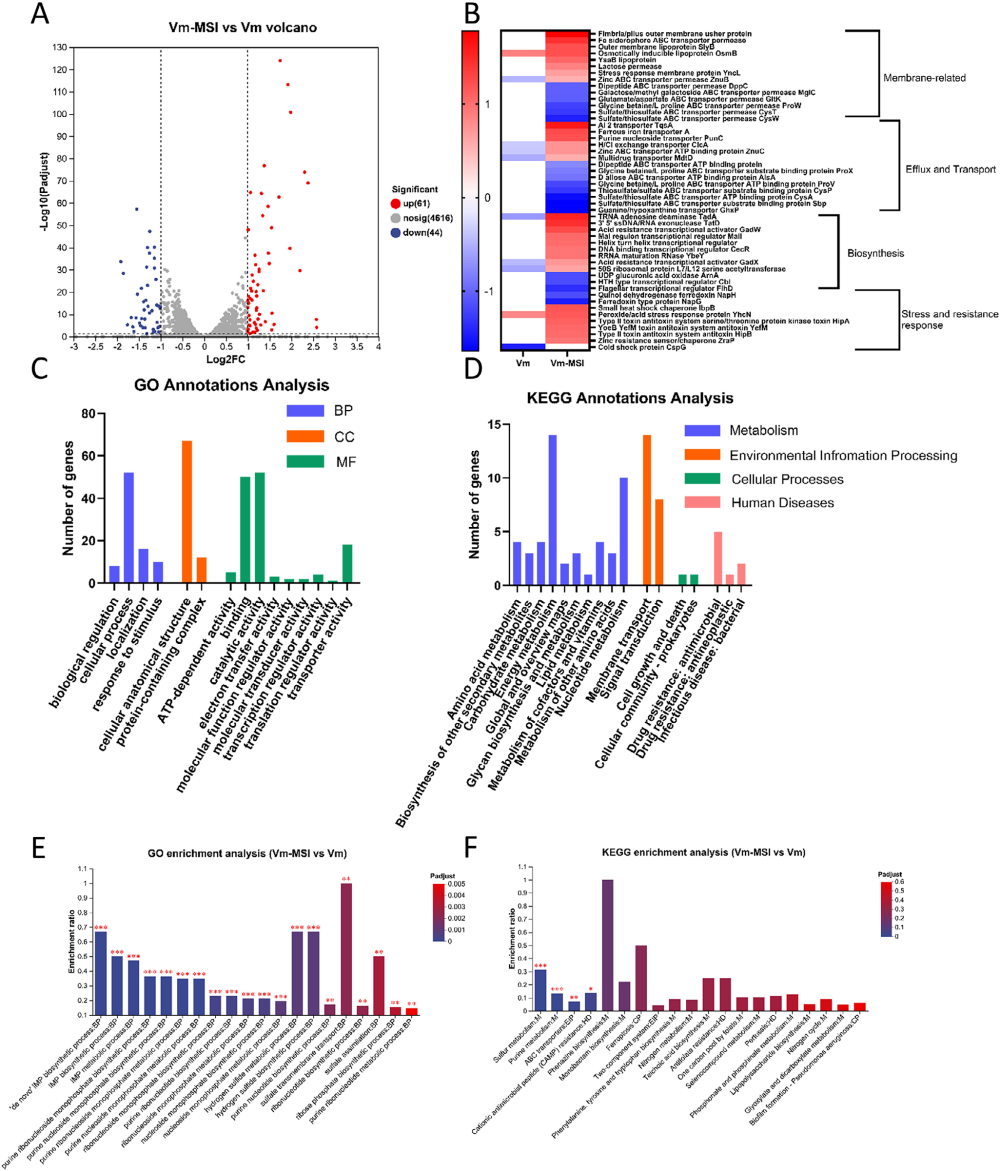
Transcriptomic profiling of *E. coli* treated with Vm‐MSI versus vancomycin. A) Volcano plot showing DEGs in *E. coli* after treatment with Vm‐MSI compared to vancomycin. Red and blue dots represent significantly upregulated and downregulated genes, respectively (|log_2_FC| ≥1, adjusted *p* < 0.05). B) Heatmap of DEGs showing log_2_ fold changes relative to the control group. Columns represent treatment groups (vancomycin and Vm‐MSI), and rows represent individual genes. Red indicates upregulation and blue indicates downregulation compared to the untreated control. C) Gene Ontology (GO) annotation of DEGs based on molecular function, cellular component, and biological process categories. D) KEGG pathway classification of DEGs into functional categories. E) GO enrichment analysis showing the top 20 significantly enriched GO terms among DEGs. (F) KEGG enrichment analysis of DEGs. n = 3 biological replicates. Source data are provided as a Source Data file.

To further elucidate the mechanism underlying Vm‐MSI's enhanced antibacterial activity against Gram‐negative bacteria, we performed transcriptomic analysis of *E. coli* treated with Vm‐MSI or vancomycin. As shown in the volcano plot (**Figure** **9**A), a total of 105 genes were significantly differentially expressed (|log_2_FC| ≥1, adjusted *p* < 0.05), including 61 upregulated and 44 downregulated genes. Hierarchical clustering analysis (Figure 9B) revealed that Vm‐MSI induced distinct transcriptional changes compared to vancomycin. Notably, the differentially expressed genes (DEGs) were organized into four functional categories: membrane‐related genes, efflux and transport systems, biosynthetic pathways, and stress and resistance response elements. These classifications reflect key cellular processes disrupted by Vm‐MSI and provide mechanistic insights into its enhanced antibacterial activity. These functional clusters closely correspond with the biological processes enriched in the subsequent GO and KEGG analyses.

Functional annotation of DEGs (Figure 9C,D) showed that Vm‐MSI primarily affected genes involved in transport, stimulus response, and core metabolic processes. GO categories such as “cellular anatomical structure,” “cellular process,” and “catalytic activity” were among the most highly represented, suggesting that Vm‐MSI broadly perturbs bacterial cellular architecture, core physiological processes, and metabolic enzyme functions. In addition, functional categories including “transporter activity” and “response to stimulus” were notably enriched, indicating that Vm‐MSI further imposes stress on membrane transport systems and activates environmental stress response pathways. KEGG classification further revealed that Vm‐MSI impacted several key bacterial pathways, including “membrane transport,” “signal transduction,” and “energy metabolism,” indicating a broader disruption of bacterial homeostasis than that induced by vancomycin alone. GO and KEGG enrichment analyses (Figure 9E,F) revealed that the DEGs were significantly associated with pathways involved in nucleotide metabolism (e.g., purine biosynthesis), transporter activity (e.g., ABC transporters), and antimicrobial resistance (e.g., CAMP resistance). These results suggest that Vm‐MSI elicits complex metabolic and stress responses in *E. coli*.

Closer inspection of representative DEGs (Figure 9B) revealed three distinct regulatory patterns. First, several genes were selectively affected by Vm‐MSI but not vancomycin, such as sulfate/thiosulfate ABC transporter protein and outer membrane lipoprotein, suggesting additional stress on membrane structure and ion homeostasis. Second, Vm‐MSI reversed the expression trends of certain vancomycin‐responsive genes, such as acid resistance regulators and zinc uptake systems, indicating that the conjugated peptide modulates bacterial adaptive strategies in a unique manner. Third, a subset of genes exhibited greater expression shifts under Vm‐MSI treatment than under vancomycin alone, including peroxide/acid stress response protein and osmotic response proteins, consistent with intensified activation of oxidative stress pathways. These results demonstrate that Vm‐MSI not only broadens the transcriptional impact of vancomycin but also redirects bacterial responses toward more severe membrane damage and redox imbalance. Altogether, these transcriptomic findings support a Vm‐MSI–specific mechanism involving enhanced disruption of bacterial defenses and offer molecular insights into its potent antimicrobial effects.

Because the transcriptomic analysis was performed at 1×MIC under shaking conditions, only limited changes were observed in biofilm‐related genes, which appeared inconsistent with the pronounced biofilm inhibition detected in our phenotypic assays. This discrepancy may be due to the fact that shaking culture conditions hinder robust biofilm formation, and that the relatively low concentration required for RNA‐seq is insufficient to capture biofilm‐specific regulatory responses. To further investigate the molecular mechanism underlying the anti‐biofilm effect, we performed qPCR analysis under static conditions, where *E. coli* ATCC25922 was incubated with vancomycin or Vm‐MSI at 8 µm for 48 h.

We focused on five classical regulators of curli‐dependent biofilm formation.^[^ ^48^, ^49^, ^50^, ^51^, ^52^ ^]^ Among them, csgD was selected as the master transcriptional regulator of the biofilm program. To better understand its regulatory network, we included two positive upstream activators (ompR and mlrA), one negative upstream regulator (rcsB), and the downstream structural gene csgA.

As shown in Figure S9 (Supporting Information), vancomycin produced only modest changes in the expression of the selected biofilm‐related genes, whereas Vm‐MSI exerted markedly stronger regulatory effects. For ompR, Vm‐MSI reduced expression to 0.3 relative to control, representing 40% greater suppression compared with vancomycin. A similar trend was observed for mlrA, where the conjugate lowered expression to 0.4, corresponding to 20% stronger inhibition than vancomycin. Notably, csgD, the master regulator of curli biogenesis, was suppressed more profoundly by Vm‐MSI (0.4) than by vancomycin (0.6), while its downstream effector csgA showed the most striking difference, with Vm‐MSI nearly abolishing expression (0.15) compared with the moderate decrease caused by vancomycin (0.8). In contrast, the negative regulator rcsB was up‐regulated to a much greater extent by Vm‐MSI (2.3 fold) than by vancomycin (1.5 fold), consistent with reinforcement of anti‐biofilm signaling.

Collectively, these results demonstrate that Vm‐MSI not only enhances the suppression of positive regulators (ompR, mlrA, csgD, csgA) but also amplifies the activation of the negative regulator (rcsB), thereby exhibiting a transcriptional profile more consistent with potent inhibition of curli‐dependent biofilm formation.

### 2.7 In Vivo Antimicrobial Activity of Vm‐MSI

**Figure 10:**
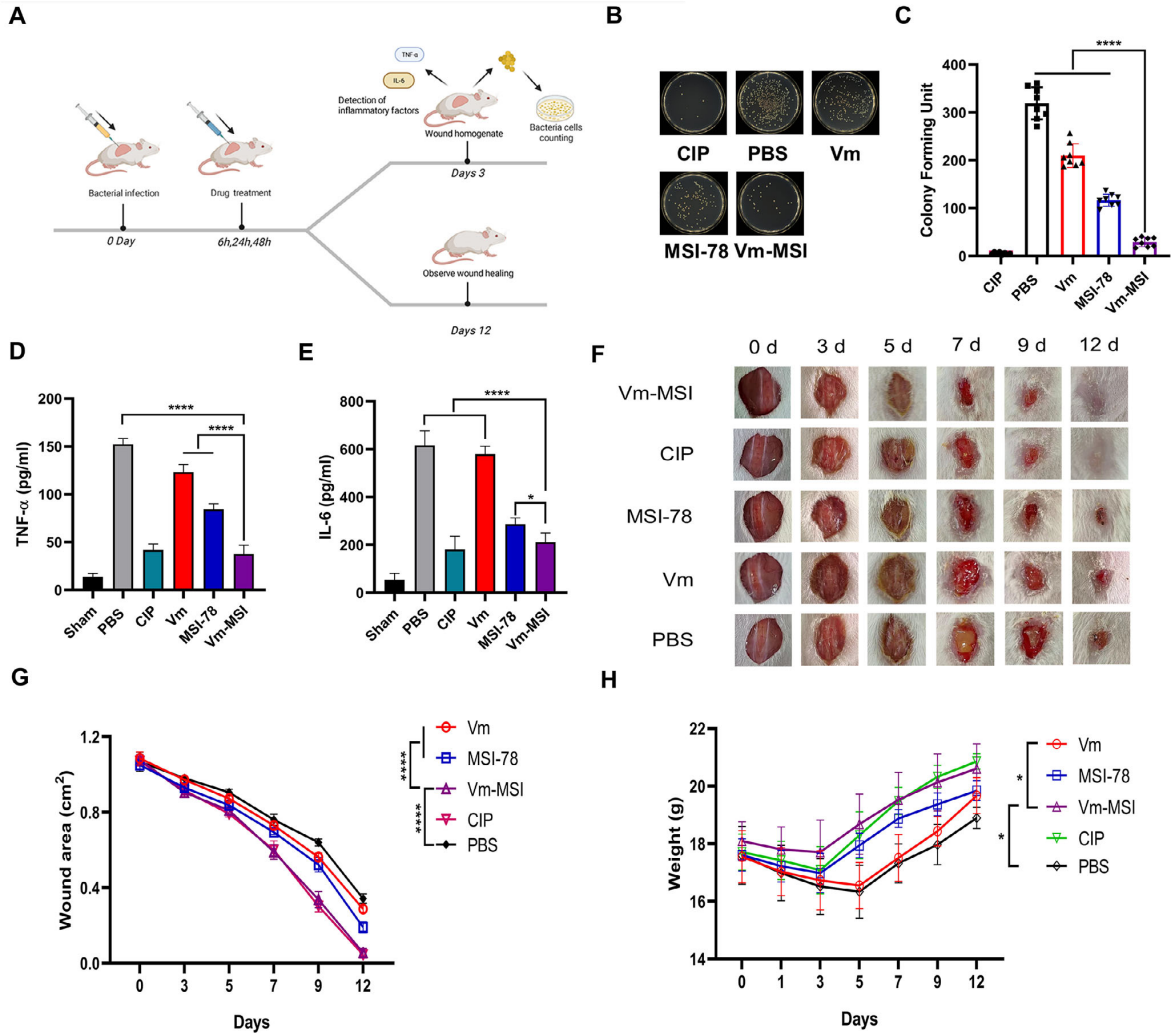
Therapeutic efficacy of Vm‐MSI in VRSA‐induced skin infection model. A) Flowchart illustrating the modeling of skin infections. The skin infection model was established by applying the vancomycin‐resistant VRSA 52 at a concentration of 1× 10^8^ CFU mL^−1^ to the wound surface of mice, with n = 8 in each experimental group. Schematic illustrations were created with BioRender.com and used with permission under a BioRender academic license. B, C) Number of bacterial colonies at the wound site after 1000 fold dilution in skin‐infected mice after 3 days of continuous drug administration (2 mg kg^−1^). D, E) Levels of pro‐inflammatory cytokines at the infected wound sites following 3 treatment applications. F) Photographs of infected wounds at days 0, 3, 5, 7, 9, and 12 for each experimental group, ciprofloxacin as a positive control, and PBS as a negative control. G) Quantification of the wound area shown in panel F. H) Body weight of mice in each experimental group recorded on days 0, 1, 3, 5, 7, 9, and 12. Statistical comparisons among multiple groups were performed using one‐way ANOVA followed by Tukey's post hoc test for pairwise comparisons, with ^*^ *p* < 0.05 and ^****^ *p* < 0.0001. n = 8 biological replicates for Figure 10C. n = 6 biological replicates for Figure 10D, E. n = 4 biological replicates for Figure 10G,H. The results are expressed as mean ± S.D. Source data are provided as a Source Data file.

To evaluate the in vivo antimicrobial efficacy of Vm‐MSI, we established two mouse infection models: a skin wound infection model and a lung infection model. Previous clinical studies have identified MRSA, VRSA, and *S. epidermidis* as the primary pathogens responsible for skin wound infections.^[^ ^53^ ^]^ Therefore, we initially investigated the therapeutic efficacy of Vm‐MSI in a VRSA‐induced skin wound infection model. Skin wound infections were induced using VRSA at a concentration of 1 × 10^8^ CFU mL^−1^. Mice were topically treated with Vm‐MSI, vancomycin, MSI‐78, and ciprofloxacin at a dosage of 2 mg kg^−1^ at 6, 24, and 48 h post‐infection. Therapeutic efficacy was evaluated by closely monitoring wound healing progress, quantifying bacterial load at the site of injury, and assessing pro‐inflammatory cytokine expression (**Figure** **10**A). Both Vm‐MSI and MSI‐78 significantly reduced bacterial load at the wound site compared to the vancomycin and PBS treatment groups. The Vm‐MSI treatment resulted in a remarkable over 90% reduction in bacterial count within the wound, which was comparable to the positive control ciprofloxacin. In contrast, MSI‐78 achieved an ≈65% reduction at the same concentration, indicating that Vm‐MSI possesses a more potent antimicrobial effect than its parent peptide MSI‐78 (Figure 10B,C). Despite VRSA being used as the bacterial strain in this experiment, vancomycin treatment also reduced bacterial load to a certain extent (≈30%). This observation is reasonable since drug resistance typically leads to decreased bactericidal efficacy rather than complete disappearance of antibacterial activity. Besides, Vm‐MSI and MSI‐78 effectively suppressed the expression of pro‐inflammatory cytokines TNF‐α and IL‐6 at the infection site, thereby alleviating excessive inflammatory responses. Notably, Vm‐MSI demonstrated superior effectiveness compared to MSI‐78 in this regard. Conversely, the vancomycin‐treated group didn't effectively decrease the levels of pro‐inflammatory cytokines TNF‐α and IL‐6, which hindered wound healing due to severe infections and inflammatory reactions (Figure 10D,E). Remarkably, Vm‐MSI significantly enhanced the rate of skin wound healing compared to the vancomycin, PBS, and MSI‐78 groups (Figure 10F,G). Like the ciprofloxacin‐positive control group, the skin wounds in the Vm‐MSI‐treated group were completely healed by 12 days post‐infection, while the wounds in the vancomycin, MSI‐78, and PBS‐treated groups remained unhealed (Figure 10F,G). Moreover, Vm‐MSI treatment most effectively reduced weight loss in mice and resulted in a quicker return to baseline weight within the first five days compared to vancomycin, PBS, MSI‐78, and ciprofloxacin groups. Throughout the entire experimental period, mice treated with Vm‐MSI maintained superior body weights than those treated with vancomycin, MSI‐78, or PBS (Figure 10H). In conclusion, these results suggest that Vm‐MSI possesses more potent in vivo antimicrobial and therapeutic effects compared to vancomycin and MSI‐78.

**Figure 11:**
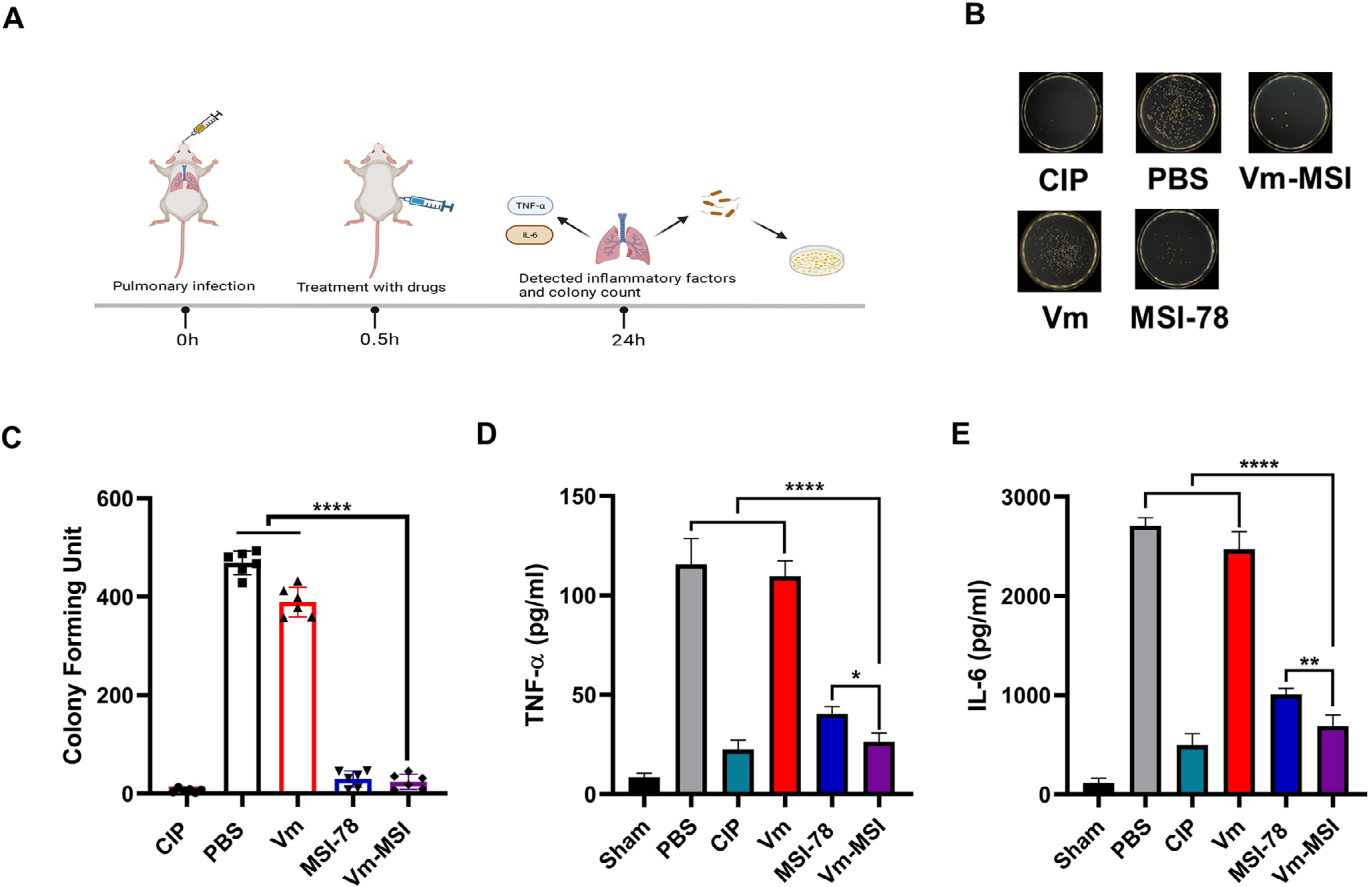
Therapeutic efficacy of Vm‐MSI in a model of MDR *A. baumannii*‐induced lung infection. A) Flowchart illustrating the modeling of lung infections. The lung infection model was established by administering multidrug‐resistant *A. baumannii* bacterial solution (50 µL, 1× 10^7^ CFU mL^−1^) via tracheal intubation into the lungs of mice. Schematic illustrations were created with BioRender.com and used with permission under a BioRender academic license. B, C) Number of colonizing bacteria in the lungs of mice. At 0.5 h post‐infection, mice were treated with Vm‐MSI (5 mg kg^−1^), vancomycin (5 mg kg^−1^), MSI‐78 (5 mg kg^−1^), or ciprofloxacin (5 mg kg^−1^) via intraperitoneal injection. After one day, the mice were euthanized, and lung tissues were collected, homogenized, diluted 10 000 fold, and inoculated to count the number of colonies. D, E) Levels of pro‐inflammatory cytokines in infected lung tissues following treatment with Vm‐MSI (5 mg kg^−1^), vancomycin (5 mg kg^−1^), MSI‐78 (5 mg kg^−1^), or ciprofloxacin (5 mg kg^−1^). The "Sham" group represents healthy mice, PBS serves as the negative control, and ciprofloxacin (5 mg kg^−1^) is the positive control. Each experimental group consisted of n = 6 mice. Statistical comparisons among multiple groups were performed using one‐way ANOVA followed by Tukey's post hoc test for pairwise comparisons, with ^*^ *p* < 0.05, ^**^ *p* < 0.01, and ^****^ *p* < 0.0001. n = 6 biological replicates for Figure 11C. n = 5 biological replicates for Figure 11D, E. The results are expressed as mean ± S.D. Source data are provided as a Source Data file.

To investigate the therapeutic efficacy of Vm‐MSI in systemic infections caused by multidrug‐resistant Gram‐negative bacteria, we established a lung infection model using multidrug‐resistant *A. baumannii*. The bacterial suspension (1 × 10^7^ CFU mL^−1^) was administered via tracheal intubation. At 0.5 h post‐infection, the mice were treated with Vm‐MSI (5 mg kg^−1^), vancomycin (5 mg kg^−1^), MSI‐78 (5 mg kg^−1^), or ciprofloxacin (5 mg kg^−1^) through a single intraperitoneal injection. After one day, the mice were euthanized, and their lung tissues were collected, homogenized, diluted, and analyzed to determine the number of colonizing bacteria and the expression of pro‐inflammatory cytokines (**Figure** **11**A).

Consistent with the findings from the skin infection model, both Vm‐MSI and MSI‐78 treatments eliminated over 95% of lung‐colonizing bacteria, which was comparable to the positive control ciprofloxacin. In contrast, vancomycin exhibited no antibacterial activity against MDR *A. baumannii*, yielding results similar to those of the PBS‐negative control group (Figure 11B, C). Furthermore, Vm‐MSI significantly reduced the levels of pro‐inflammatory cytokines in lung tissues. The levels of pro‐inflammatory cytokines TNF‐α and IL‐6 in the Vm‐MSI treatment group were similar to those in the ciprofloxacin‐positive control group and significantly lower than those observed in the MSI‐78, vancomycin, and PBS‐negative control groups (Figure 11D,E). In conclusion, these results demonstrate that Vm‐MSI exhibits excellent therapeutic efficacy in systemic infections and drug‐resistant Gram‐negative bacterial infections.

## 3 Discussion

To address the growing issue of vancomycin resistance and its limited antimicrobial spectrum, structural modification of vancomycin has become a widely pursued research direction. We have discussed the limitations of two commonly used modification approaches in the introduction section. Here, we introduce a novel modification strategy that employs linkers as chemical bridges to conjugate vancomycin with AMPs, yielding a promising antimicrobial agent. To achieve this, we selected four AMPs with distinct mechanisms of action that complement vancomycin. Next, we conjugated the cysteine‐modified AMP with the vancomycin‐linker complex. This approach offers the advantage of maximizing synthesis yield for both the peptide and vancomycin, while limiting the final reaction to two primary components, thereby reducing by‐product formation. Moreover, the linker preserves the intrinsic active groups of vancomycin and AMP, while introducing a maleimide group to vancomycin that can undergo a highly selective, rapid Michael addition reaction with the thiol group in the cysteine residue under mild conditions.^[^ ^4^, ^16^, ^54^ ^]^ However, maleimide–thiol linkages are known to be susceptible to Retro‐Michael reactions, particularly under reducing conditions, which may limit their long‐term stability in certain biological environments. Despite this limitation, we employed this strategy due to its high efficiency, aqueous compatibility, and widespread use in bioconjugation, in line with the proof‐of‐concept nature of this study. Finally, we synthesized four distinct vancomycin‐AMP conjugates with the aim of identifying effective candidates capable of overcoming vancomycin resistance and addressing the urgent need to combat Gram‐negative bacterial infections.

Through antimicrobial and anti‐biofilm screening, along with hemolysis evaluation, we identified Vm‐MSI as a potent lead compound. This study highlights Vm‐MSI's broad‐spectrum antimicrobial activity and its high efficacy against multidrug‐resistant VRSA strains. Notably, Vm‐MSI demonstrated strong antibacterial action against VRSA at a concentration of 2.21 µm (Table 1). However, the dense outer membranes of Gram‐negative bacteria remain a major barrier to effective antibiotic delivery, significantly limiting the activity of vancomycin, erythromycin, linezolid, fosfomycin, and rifampicin against these pathogens.^[^ ^17^ ^]^ Vm‐MSI not only effectively delayed the development of vancomycin resistance in VRSA but also exhibited strong activity against key Gram‐negative pathogens, including multidrug‐resistant *A. baumannii*, *P. aeruginosa*, and *K. pneumoniae*, which are notorious for causing persistent clinical infections. Vm‐MSI achieved MICs between 1.10 and 4.42 µm across these strains. In an extensive antimicrobial efficacy evaluation against 11 common Gram‐positive and 18 Gram‐negative pathogens, Vm‐MSI showed a more than 20.18 fold improvement in antimicrobial activity compared to vancomycin alone. These results underscore Vm‐MSI's potential as an effective treatment for vancomycin‐resistant and drug‐resistant Gram‐negative infections.

Bacteria have developed robust defense mechanisms, such as biofilm formation and the ability to produce persister cells.^[^ ^19^ ^]^ This forms the basis of chronic infections that are difficult to eradicate, potentially leading to significant safety concerns.^[^ ^20^ ^]^ Biofilm formation progresses through four stages: adhesion, growth, differentiation, and colonization.^[^ ^21^ ^]^ The mucus matrix, composed of polysaccharides, proteins, DNA, and other compounds produced during bacterial colonization, can inhibit antibiotic penetration and reduce their killing effectiveness.^[^ ^20^, ^35^, ^55^, ^56^ ^]^ As mentioned above, bacterial motility enables strong migration,^[^ ^33^ ^]^ facilitating biofilm formation that allows bacteria to evade immune clearance and resist antibiotics, ultimately leading to the formation of persistent cells. Once survival pressures decrease, these dormant, antibiotic‐resistant bacteria can reactivate, causing recurrent infections. Vm‐MSI, with its ability to inhibit bacterial motility and rapid bactericidal action, effectively slows bacterial adhesion and colonization, destroying most bacteria before biofilm formation can begin (Figures 2 and 3). Additionally, the amphiphilic nature of MSI‐78 allows it to penetrate biofilm matrices, targeting bacteria within biofilms and eliminating both biofilm structures and resident cells. Vm‐MSI eliminated 99% of resident bacteria under biofilms at a concentration of 8 µm, an efficacy surpassing that of vancomycin (Figure 4). Moreover, at this concentration, Vm‐MSI showed superior anti‐biofilm and anti‐persister cell activity against multidrug‐resistant strains compared to MSI‐78 alone.

Most conventional antibiotics operate through a single target and mechanism, which gives bacteria more opportunities to develop mutations at specific sites, facilitating the development of antibiotic resistance.^[^ ^19^, ^57^ ^]^ In contrast, AMPs employ complex mechanisms of action and act quickly to kill bacteria, making it difficult for bacteria to develop resistance.^[^ ^55^ ^]^ Therefore, combining the two agents has become a common approach to prevent drug resistance and rapidly eradicate bacteria.^[^ ^12^, ^58^ ^]^ Our results indicate that Vm‐MSI demonstrates synergistic antimicrobial effects with polymyxin B, ciprofloxacin, and gentamicin (Figure 5). Notably, the combination of Vm‐MSI with polymyxin B enhanced antibacterial activity by 8–32 times, and significant synergistic effects were observed with other antibiotics as well. This demonstrates that Vm‐MSI, when combined with other antibiotics, can effectively eliminate drug‐resistant and Gram‐negative bacteria. Additionally, while bacteria developed resistance to ciprofloxacin and vancomycin after less than 20 generations of continuous culture at sub‐MIC, few bacteria developed stable resistance to Vm‐MSI and MSI‐78 (Figure 2). The low frequency of resistance to the multi‐mechanism Vm‐MSI underscores its considerable potential for clinical application.

The diverse mechanisms of AMP enable them to synergize effectively with antibiotics. To investigate this in depth, we focused on two key antibacterial mechanisms common to AMP: membrane disruption and the induction of bacterial oxidative stress. Our findings show that Vm‐MSI significantly compromises the structural and functional integrity of the bacterial cell membrane by altering its permeability, fluidity, and degree of depolarization, indicating that membrane disruption is a crucial aspect of Vm‐MSI's antibacterial mechanism (Figures 6 and 7). As previously mentioned, ROS are natural metabolites in biological processes; their accumulation can cause oxidative stress, ultimately damaging bacterial DNA, proteins, and other biomolecules. This mechanism is commonly employed by certain AMPs and antibiotics, such as aminoglycosides and quinoline antibiotics.^[^ ^59^ ^]^ While vancomycin does not significantly induce ROS production, Vm‐MSI was found to induce ROS generation across various concentrations. At higher concentrations, the level of ROS induced by Vm‐MSI was more than double that of MSI‐78 at the same concentration. Additionally, the use of ROS scavengers significantly reduced Vm‐MSI's bactericidal activity, confirming that ROS generation and accumulation‐induced oxidative stress is one of its key mechanisms of action (Figure 8). To further elucidate the mechanism underlying Vm‐MSI's enhanced antibacterial activity, we performed transcriptomic profiling of *E. coli* treated with Vm‐MSI or vancomycin. Compared with vancomycin, Vm‐MSI induced broader and more distinct transcriptional changes, particularly affecting genes related to membrane structure, transport systems, stress response, and metabolic regulation. Functional enrichment analysis revealed that Vm‐MSI significantly influenced key bacterial pathways, including ABC transporters, signal transduction, energy metabolism, CAMP resistance, and purine biosynthesis. Several membrane‐associated and redox‐responsive genes were uniquely regulated by Vm‐MSI, and some vancomycin‐responsive genes showed reversed or amplified expression trends under Vm‐MSI treatment. These results suggest that Vm‐MSI exerts its antibacterial effect through multifaceted physiological stress, involving enhanced disruption of membrane homeostasis, interference with stress adaptation mechanisms, and alteration of essential metabolic pathways. This transcriptomic evidence provides mechanistic insights into the superior antibacterial performance of Vm‐MSI against Gram‐negative bacteria (Figure 9). Furthermore, we evaluated the inherent antibacterial mechanism of vancomycin in the antimicrobial blockade experiment using D‐Ala‐D‐Ala. As expected, Vm‐MSI retains vancomycin's intrinsic bactericidal properties, establishing a strong foundation for Vm‐MSI as a broad‐spectrum, highly effective antibacterial agent (Figure 7). Based on these mechanistic findings, we next sought to determine whether Vm‐MSI's enhanced antibacterial activity could be recapitulated in vivo.

Antibiotic resistance can turn simple wound infections, which account for over 4% of all emergency room visits in the United States, into life‐threatening conditions. Chronic wounds, affecting ≈6.5 million people in the U.S., are also particularly vulnerable to bacterial infections, often involving biofilms.^[^ ^56^ ^]^ Our study demonstrated that Vm‐MSI is highly effective in treating VRSA‐infected skin wounds, using a therapeutic model of drug‐resistant bacterial infection in mice. After three doses, Vm‐MSI cleared over 90% of VRSA at the wound site, matching the efficacy of ciprofloxacin, a first‐line treatment for skin infections. Additionally, Vm‐MSI reduced excessive inflammation, accelerating skin wound healing and supporting weight recovery in infected mice (Figure 10).

Currently, multidrug‐resistant Gram‐negative bacteria pose a major threat to hospitalized patients, with associated mortality rates ranging from 30% to 70%.^[^ ^60^ ^]^ These pathogens have become an increasingly significant global healthcare concern.^[^ ^61^ ^]^ The estimated global incidence of *A. baumannii* infections is ≈1 million cases annually. The World Health Organization has acknowledged the growing importance of this species, listing carbapenem‐resistant *A. baumannii* (CRAB) as one of the priority pathogens in the ′Global priority list of antibiotic‐resistant bacteria to guide research, discovery, and development of new antibiotics, 2017′. This pathogen primarily affects immunocompromised patients, causing pneumonia and bloodstream infections with a high mortality rate.^[^ ^62^ ^]^ The primary challenge in treating infections caused by these Gram‐negative bacteria is their frequent drug resistance, particularly in persistent pneumonia infections, which often result in severe complications such as respiratory failure, bacteremia, shock, and acute respiratory distress syndrome (ARDS).^[^ ^63^ ^]^ To address this, we developed a mouse lung infection model using multidrug‐resistant *A. baumannii*. The results demonstrated that Vm‐MSI effectively cleared over 95% of multidrug‐resistant *A. baumannii* colonized in the lungs within 24 h after administration. It also reduced elevated pro‐inflammatory cytokines in lung tissue, confirming its potential for treating systemic infections in vivo (Figure 11). In summary, these findings suggest that Vm‐MSI holds promise for clinical application in the treatment of both common skin infections (such as burns, diabetic foot ulcers, and certain dermatitis conditions) and systemic infections, including drug‐resistant pneumonia and sepsis.

In conclusion, Vm‐MSI represents a promising solution for challenging infections. It shows promising activity against VRSA and demonstrates potent, broad‐spectrum antibacterial activity against multidrug‐resistant strains, biofilms, and persister cells. We confirmed its safety profile, low potential for resistance development, and synergistic antibacterial effects. Moreover, Vm‐MSI showed impressive therapeutic efficacy in treating surface wounds and systemic infections caused by multidrug‐resistant bacteria. Vm‐MSI stands as a strong candidate for combating infections and addressing bacterial resistance.

## 4 Experimental Section

### Animals and Ethical Statement

All animal experiments were approved by the Animal Ethics Review Committee of the Yantai Institute of Coastal Zone Research, Chinese Academy of Sciences. The experimental protocols complied with the Animal Welfare Law. Female BALB/c mice (6–8 weeks old, 18–20 g) were obtained from Ziyuan Laboratory Animal Technology Co., Ltd., China. The animals were housed with unrestricted access to food and water under a 12 h light/dark cycle, with environmental conditions maintained at a temperature of 20–26 °C and relative humidity of 40–70%.

### Synthesis of Vancomycin‐AMP Conjugates

The synthesis method was based on a previous report with slight modifications.^[^ ^22^ ^]^ Four AMPs were synthesized by GL Biochem (Shanghai) Ltd. (Shanghai, China) using the Fmoc solid‐phase peptide synthesis method, including Omiganan (ILRWPWWPWRRK‐NH_2_), MSI‐78 (GIGKFLKKAKKFGKAFVKILKK‐NH_2_), Bac‐7 (RRIRPRPPRLPRPRPRP), and Pleurocidin (GWGSFFKKAAHVGKHVGKAALTHYL‐NH_2_). To facilitate the synthesis of subsequent conjugates, an additional cysteine residue was introduced at the N‐terminus of each peptide.

Vm‐SMCC was prepared by stirring vancomycin (MCE, US) with Sulfo‐SMCC linker (Bide Pharmatech Ltd., China) at a molar ratio of 1:1 at room temperature for 3 h, with reaction progress monitored by analytical reverse‐phase high‐performance liquid chromatography (RP‐HPLC, 1260, Agilent, US). The product was identified by LC‐MS (6120, Agilent, US) and purified using preparative RP‐HPLC (HT7050A, Huitongsepu Ltd., China). Finally, Vm‐SMCC and AMP were reacted in a PBS system at a molar ratio of 1:1 at room temperature for 2 h. The reaction progress was similarly monitored by analytical RP‐HPLC, and the products were identified and purified using LC‐MS and preparative RP‐HPLC, respectively.

### Circular Dichroism Spectroscopy

CD spectroscopy was performed to examine the secondary structure of MSI‐78 and Vm‐MSI. Peptides were dissolved at a final concentration of 200 µg mL^−1^ in either deionized water or a 60 mm SDS aqueous solution. CD spectra were recorded at 25 °C using a Chirascan CD spectropolarimeter (Applied Photophysics, UK) with a 1 mm path length quartz cuvette. The scanning wavelength range was set from 180 to 260 nm, with a bandwidth of 1 nm and a scan speed of 100 nm min^−1^. Each spectrum was averaged from three consecutive scans and corrected by subtracting the corresponding solvent baseline. Results were expressed as ellipticity in millidegrees (mdeg).

### Hemolytic Activity Assay

Human erythrocytes were collected from healthy volunteers, and informed written consent was obtained from all participants prior to the study. Following previously described methods,^[^ ^64^ ^]^ freshly collected human and mouse blood samples were mixed with Alsever's solution (Biosharp, China) and centrifuged at 1200 rpm for 3 min. The resulting pellet was washed twice with saline and resuspended in PBS at a concentration of 10⁷–10⁸ cells mL^−1^. The diluted erythrocyte suspensions were then mixed with vancomycin‐AMP conjugates dissolved in saline to achieve final concentrations of 4, 8, 16, 32, and 64 µM. The mixtures were incubated at 37 °C for 30 min. After incubation, the samples were centrifuged at 1000 rpm for 5 min, and the absorbance of the supernatant was measured at 540 nm. Saline was used as the negative control, and 1% Triton X‐100 (Beyotime, China) served as the positive control. The percentage of hemolysis was calculated using the formula: Hemolysis percentage (%) = A _sample_‐A _negative control_/A _positive control_ × 100%.

### Minimum Inhibitory Concentration Assay

The 2 fold broth microdilution method was performed following previously established protocols.^[^ ^65^, ^66^ ^]^ A total of 29 strains of human pathogenic microorganisms (11 Gram‐positive and 18 Gram‐negative) were included in the assay. Standard strains were maintained in our laboratory, and clinical isolates were obtained from the Fourth Affiliated Hospital of Soochow University. Bacteria were cultured to the logarithmic growth phase in Mueller–Hinton (MH) broth at 37 °C and diluted to 2 × 10⁵ CFU mL^−1^. Gradient dilutions of the drugs (vancomycin, AMPs, and vancomycin‐AMP conjugates) were prepared in 96‐well plates, with 50 µL of each drug solution added per well. For the vancomycin and AMP mixture, 25 µL of vancomycin was combined with 25 µL of AMP at the corresponding monomer gradient concentration, resulting in a total volume of 50 µL. Next, 50 µL of the diluted bacterial suspension was added to each well, and the plates were incubated at 37 °C with shaking at 110 rpm for 18 h. Following incubation, bacterial growth was assessed by measuring absorbance at 600 nm. The MIC was defined as the lowest concentration at which no bacterial growth was detected.

### Live/Dead Bacteria Staining Assay

Following the methodology described in previous literature,^[^ ^18^ ^]^ VRSA 52 and *E. coli* ATCC25922 were cultured to logarithmic growth in MH medium at 37 °C with shaking at 200 rpm. The cultures were centrifuged and resuspended in PBS buffer, a process repeated three times. The final bacterial concentration was diluted to 1× 10^8^ CFU mL^−1^ with PBS buffer. Vancomycin, MSI‐78, and Vm‐MSI were incubated at 37 °C with shaking at 200 rpm for 1 h to achieve a final concentration of 4 µm. After 1 h, 3 µL of SYTO9 (Invitrogen, US) and PI (Invitrogen, US) fluorescence dyes were added, with final concentrations of 0.83 and 5 mm, respectively. The mixture was incubated at 37 °C with shaking at 200 rpm for an additional 30 min. Following incubation, the samples were centrifuged and resuspended in PBS buffer to wash out the fluorescence dyes and drugs. The sample was mixed with the fixative, mounted on a slide, and imaged using a confocal microscope (AIR HD25, Nikon, Japan). For consistency, images were captured at the same laser intensity for each experiment. Image processing was performed with NIS‐Elements Viewer 4.20 software, focusing on the relative intensities of red and green fluorescence. Quantitative analysis of live and dead bacteria was carried out using ImageJ software.

### Bacterial Killing Kinetics Assay

With slight modifications to previously established methods,^[^ ^66^, ^67^ ^]^ *S. aureus* CMCC26003 and *E. coli* ATCC25922 were cultured to logarithmic growth in MH broth at 37 °C. The cultures were then diluted to 1 × 10^6^ CFU mL^−1^ with fresh MH medium, and the drugs (vancomycin, MSI‐78, and Vm‐MSI) were added to achieve a final concentration of 4 µm. The mixtures were incubated at 37 °C, and samples of 50 µL were taken at 0, 15, 30, 60, 120, and 180 min. Each sample was diluted 1000 times with fresh MH medium, and 50 µL of the dilution was evenly spread on MH agar plates. The plates were then incubated at 37 °C for 18 h, after which colonies were counted.

### Anti‐Persister Cells Assay

Following previously established methods,^[^ ^18^, ^68^ ^]^ *S. aureus* CMCC26003 was cultured to logarithmic growth in MH broth at 37 °C and diluted to 1 × 10^8^ CFU mL^−1^ with fresh MH broth. A final concentration of 20× MIC of ciprofloxacin (Sigma–Aldrich, US) was added to 1 mL of the bacterial suspension. The mixture was incubated at 37 °C with shaking at 200 rpm for 8 h. Bacterial samples were collected at 0, 2, 4, 6, and 8 h and quantified by plating. The remaining bacterial solution was centrifuged and resuspended in PBS to remove ciprofloxacin, followed by treatment with vancomycin, MSI‐78, or Vm‐MSI, each dissolved in fresh MH broth. The mixtures were incubated at 37 °C with shaking at 200 rpm for up to 48 h. Bacterial samples were collected at 24 and 48 h, diluted, plated, and then incubated at 37 °C for 18 h for colony counting.

### Antimicrobial Resistance Development Assay

The evolution of bacterial resistance to vancomycin, ciprofloxacin, AMPs, and their conjugates was assessed following a previously described method^[^ ^69^ ^]^ with slight modifications. *S. aureus* CMCC26003 and *E. coli* ATCC25922 in the logarithmic growth phase were diluted and added to fresh MH medium containing 1/4× MIC concentrations of the drugs. The cultures were incubated at 37 °C with shaking at 200 rpm for 12 h, and this process was repeated for successive bacterial generations. The MIC of each culture was determined every five generations using a serial dilution method in 96‐well plates. Starting from the 10th generation, the drug concentration was increased to 1/2× MIC to heighten bacterial survival pressure and further drive the development of resistance.

### Salt Ion Stability Assay

In accordance with the methods from previous literature,^[^ ^70^ ^]^ the salt ion stability of vancomycin‐AMP conjugates was evaluated under physiological conditions using VRSA 52 and *E. coli* ATCC25922. The bacteria were cultured to logarithmic growth in MH medium at 37 °C and then diluted in fresh MH medium to a concentration of 2 × 10^5^ CFU mL^−1^. In 96‐well plates, the vancomycin‐AMP conjugates were diluted in MH medium containing physiological salt ion concentrations (NaCl 150 mm, KCl 4.5 mm, CaCl_2_ 2 mm, MgCl_2_ 1 mm, ZnCl_2_ 8 µm, FeCl_3_ 4 µm, and NH_4_Cl 6 µm, Sinopharm Chemical Reagent Co., Ltd, China). MH medium without salt ions served as a blank control. A volume of 50 µL of the diluted vancomycin‐AMP conjugates was added to each well, followed by the addition of 50 µL of the diluted bacterial solution. The subsequent steps were performed as per the MIC experiments.

### Swimming Motility Experiment

This experiment was adapted from the method of Shi et al. ^[^ ^42^ ^]^ with slight modifications. A specific culture medium was prepared, consisting of tryptone (10 g L^−1^, Oxoid, US), NaCl (10 g L^−1^, Sangon Biotech, China), yeast extract (5 g L^−1^, Sangon Biotech, China), and agar (0.3%, neoFroxx, Germany), followed by sterilization. Vancomycin, MSI‐78, and Vm‐MSI were added to the medium at final concentrations of 0.5, 1, and 2 µM after cooling. PBS was used as a negative control, while polymyxin B (Sigma–Aldrich, US) served as a positive control. *S. aureus* CMCC26003 and *E. coli* ATCC25922 were cultured to the logarithmic growth phase, then diluted to 10⁶ CFU mL^−1^ using fresh MH medium. Subsequently, 5 µL of the dilution was placed onto the surface of the cooled solid culture medium. The plates were incubated at 37 °C for 24 h. After incubation, the plates were photographed, and colony areas were quantified using ImageJ software.

### Antibiofilm Assay

Following the methods described in previous studies,^[^ ^64^, ^69^ ^]^ bacteria were cultured to the logarithmic growth phase and diluted to 10⁷ CFU mL^−1^ using fresh MH medium. The diluted bacterial suspension (100 µL per well) was added to a 96‐well plate and incubated at 37 °C for 48 h to allow biofilm formation. After incubation, the wells were washed three times with PBS buffer, and 100 µL of vancomycin, AMPs, or their conjugates at final concentrations of 1, 2, 4, and 8 µM was added. PBS buffer was used as a negative control. The plate was incubated at 37 °C for 24 h, then washed three more times with PBS buffer and fixed with methanol. Finally, the biofilms were stained with crystal violet (Solarbio, China), and optical density (OD) was measured at a wavelength of 595 nm using a UV spectrophotometer.

To evaluate the biofilm inhibition capacity of the peptides, the bacterial suspension diluted to 10⁷ CFU mL^−1^ was mixed with vancomycin, MSI‐78, and Vm‐MSI at final concentrations of 0.5, 1, 2, and 4 µM. The mixture was incubated in a 96‐well plate for 48 h. Subsequent steps, including washing, fixation, staining, and OD measurement, were performed as described in the biofilm eradication assay.

BR (%) = 100% × F_drug_/F_0_, where PBS (F_0_) is the negative control and its measured value is considered as the maximum biofilm residue, Biofilm Retention%, BF% is the percentage of biofilm residue, and F_drug_ is the absorbance value of the administered treatment group.

BF (%) = 100% × F_drug_/F_0_, where PBS (F_0_) is the negative control and its measurement is considered as the maximum biofilm formation, Biofilm Formation%, BF% is the percentage of biofilm formation, and F_drug_ is the absorbance value of the administered treatment group.

### TTC Assay

Following the experimental method described in reference,^[^ ^36^ ^]^ VRSA 52, *E. coli* ATCC25922, and MDRP5 were cultured to the logarithmic growth phase and diluted to 10⁷ CFU mL^−1^ with fresh MH medium. A 100 µL aliquot of the diluted bacterial suspension was added to each well of a 96‐well plate and incubated at 37 °C for 24 h. After incubation, the wells were washed three times with PBS buffer, and 90 µL of drug solutions (vancomycin, MSI‐78, Vm‐MSI) diluted in MH medium were added at final concentrations of 1, 2, 4, and 8 µM. Subsequently, 10 µL of TTC solution (Sigma–Aldrich, US) was added to each well to achieve a final concentration of 0.05%, and PBS buffer was used as a negative control. The plate was incubated overnight at 37 °C. After incubation, planktonic bacteria were removed, and the reduced TTC formed was dissolved in methanol. The absorbance was then measured at 500 nm to quantify biofilm formation.

For the TTC biofilm inhibition experiment, 50 µL of the diluted bacterial suspension was added to each well of a 96‐well plate, followed by 40 µL of drug solutions (vancomycin, MSI‐78, and Vm‐MSI) at final concentrations of 0.5, 1, 2, and 4 µM. Next, 10 µL of TTC solution (final concentration of 0.05%) was added to each well. After overnight incubation, planktonic bacteria were discarded, and the reduced TTC was fully dissolved in methanol. The absorbance was measured at 500 nm for quantification.

### Biofilm Resident Bacteria Assay

VRSA 52, *E. coli* ATCC25922, and MDRP5 were cultured to the logarithmic growth phase following a previously described method^[^ ^56^ ^]^ with slight modifications. The cultures were then diluted to 1 × 10⁷ CFU mL^−1^ using fresh MH medium, and 100 µL of the diluted bacterial suspension was added to each well of a 96‐well plate. The plate was incubated statically at 37 °C overnight. After incubation, planktonic bacteria were discarded, and the wells were washed three times with PBS buffer. Fresh MH medium containing 20× MIC ciprofloxacin was added to each well, and the plate was incubated statically at 37 °C for 24 h. The planktonic bacteria were discarded, and the wells were washed three more times with PBS buffer. Biofilm‐resident bacteria were harvested by scraping the wells and subjecting the samples to 10 min of sonication in 100 µL of culture medium.

Subsequently, the biofilm‐resident bacteria were exposed to vancomycin, MSI‐78, and Vm‐MSI at final concentrations of 1, 2, 4, and 8 µM, with PBS buffer used as a negative control. The plate was incubated at 37 °C for 24 h. Finally, 50 µL of the culture medium from each well was collected, diluted, and plated for colony counting.

### CLSM Assay of Biofilm‐Resident Bacteria

VRSA 52 and *E. coli* ATCC25922 were cultured in MH medium at 37 °C with shaking at 200 rpm until reaching the logarithmic growth phase. The cultures were then diluted to 1 × 10^7^ CFU mL^−1^ with fresh MH medium and seeded into glass‐bottom culture dishes, followed by static incubation at 37 °C for 48 h to allow biofilm formation. After incubation, the biofilms were gently washed three times with PBS to remove planktonic bacteria, and subsequently treated with vancomycin, MSI‐78, or Vm‐MSI (8 µm) for 4 h under static conditions. After drug treatment, 3 µL of SYTO9 (Invitrogen, US) and PI (Invitrogen, US) dyes were added at final concentrations of 0.83 and 5 mm, respectively, and incubated at 37 °C for 30 min in the dark. The samples were washed three times with PBS to remove excess dyes, and then imaged using a confocal microscope (AIR HD25, Nikon, Japan). For consistency, images were acquired under identical laser intensity settings for each experiment. Image processing was performed with NIS‐Elements Viewer 4.20 software, and the relative intensities of green (live cells) and red (dead cells) fluorescence were analyzed using ImageJ software.

### Synergy Antimicrobial Assay

Following the previous experimental methods,^[^ ^71^ ^]^ a checkerboard assay was conducted to evaluate whether the vancomycin‐AMP conjugate exhibited a synergistic antimicrobial effect when combined with four antibiotics that have different mechanisms of action. The interaction between the two antimicrobial agents was classified as synergistic, antagonistic, additive, or irrelevant based on the calculation of the FIC value, using the formula: FIC = F_MICA_ + F_MICB_ = A/MIC_A_+B/MIC_B_. Here, A and B represent the concentrations at the optimal combination point for the two drugs, while MICA and MICB represent the MIC for each drug when used alone. The criteria for interpretation were as follows: synergistic if FIC ≤ 0.5, antagonistic if FIC > 2, additive if 0.5 < FIC ≤ 1, and irrelevant if 1 < FIC ≤ 2.

### Scanning Electron Microscopy Assay

In accordance with the experimental methods described in previous literature,^[^ ^42^ ^]^ VRSA 52 and *E. coli* ATCC25922 were cultured to the logarithmic growth phase, then centrifuged and washed three times with PBS buffer. The drugs (vancomycin, MSI‐78, and Vm‐MSI) were added to each sample at a final concentration of 4 µm, with polymyxin B serving as a positive control and PBS buffer as a negative control. The samples were incubated at room temperature for 2 h. After drug administration, the samples were fixed in 2.5% glutaraldehyde (Biosharp, China) for 5 h. Subsequently, the samples underwent dehydration using graded ethanol (30%, 50%, 70%, 90%, and 100%). The dehydrated samples were then dried, gold‐coated, and imaged using an S‐4800 SEM instrument (Hitachi, Japan).

### Bacterial Membrane Permeability Assay

In accordance with our previously established experimental method,^[^ ^69^ ^]^ slightly modified for Gram‐positive bacteria, *S. aureus* CMCC26003 and VRSA 52 were cultured to the logarithmic growth stage in MH medium at 37 °C with shaking at 200 rpm. The cultures were then centrifuged at 7000 rpm and washed three times with PBS buffer, after which they were diluted to a concentration of 1 × 10^8^ CFU mL^−1^ with PBS buffer. To assess membrane permeability, PI dye was added to the diluted bacterial solution at a final concentration of 40 µg mL^−1^ and incubated at 37 °C for 30 min in the dark. Following this, 50 µL of the stained bacterial solution was added to a 96‐well black plate, and 50 µL of the drugs (vancomycin, MSI‐78, and Vm‐MSI) was added at final concentrations of 1, 2, 4, and 8 µm. The plates were incubated at 37 °C, protected from light, for 1 h. After incubation, fluorescence was measured using a Tecan Infinite M1000 PRO microplate reader (Tecan, Switzerland) with excitation at 535 nm and emission at 615 nm. For controls, 10% Triton X‐100 was used as a positive control, indicating 100% bacterial membrane permeability, while PBS buffer served as a negative control, indicating 0% membrane permeability. The results were then converted to percentages of inner membrane permeability using the following formula:

Inner Membrane Permeability (%) = (FI_drug_ ‐ FI_PBS_) / (FI_Triton X‐100_ ‐ FI_PBS_) × 100%, where FI_drug_ is the fluorescence intensity observed in the administered group at a given concentration, FI_PBS_ is the fluorescence intensity in the PBS negative control group, and FI_Triton X‐100_ is the fluorescence intensity in the 10% Triton X‐100 (v/v) positive control group.

For Gram‐negative bacteria, *E. coli* ATCC25922 was cultured to the logarithmic growth phase in MH medium, then centrifuged at 7000 rpm and washed three times with PBS buffer. The bacterial pellet was finally diluted to a concentration of 1× 10^8^ CFU/mL with PBS buffer. In 96‐well black plates, 80 µL of the diluted bacterial solution was added to each well, followed by the addition of 10 µm N‐phenyl‐1‐naphthylamine (NPN, Sigma–Aldrich, US) dye to achieve the final concentration. The plates were incubated at 37 °C, protected from light, for 30 min. After incubation, 10 µL of the drugs (vancomycin, MSI‐78, and Vm‐MSI) was added to each well to achieve final concentrations of 1, 2, 4, and 8 µm. Additionally, 100 µg mL^−1^ polymyxin B was used as a positive control, indicating 100% bacterial membrane permeability, while PBS buffer served as a negative control, indicating 0% membrane permeability. The results were then converted to percentages of inner membrane permeability using the following formula:

Outer Membrane Permeability (%) = (FI_drug_ – FI_PBS_) / (FI_PMB_ – FI_PBS_) × 100%, where FI_drug_ is the fluorescence intensity observed in the administered group at the given concentration, FI_PBS_ is the fluorescence intensity in the PBS negative control group, and FI_PMB_ is the fluorescence intensity in the 100 µg/ml polymyxin B positive control group.

### Bacterial Membrane Fluidity Assay

Following the experimental method outlined in previous literature^[^ ^40^ ^]^ with slight modifications, *S. aureus* CMCC26003, VRSA 52, and *E. coli* ATCC25922 were cultured to the logarithmic growth phase, then centrifuged and washed three times with PBS buffer. The bacterial pellet was diluted to a concentration of 5 × 10^7^ CFU mL^−1^ and incubated at 37 °C in the presence of 10 µm Laurdan dye (MCE, US), protected from light, for 30 min. After incubation, various concentrations of drugs (vancomycin, MSI‐78, and Vm‐MSI) were added to the wells, with 20% Triton X‐100 serving as a positive control and PBS buffer as a negative control. The incubation continued at 37 °C, protected from light, for 1 h. Fluorescence was recorded using a Tecan Infinite M1000 PRO microplate reader (Tecan, Switzerland), with excitation at 350 nm and emission wavelengths at 435 and 490 nm. Laurdan GP values were calculated using the following formula:

(1) $$\mathrm{Laurdan}\;\mathrm{GP}=\left(FI_{435}-FI_{490}\right)/\left(FI_{435}+FI_{490}\right)$$

### Bacterial Membrane Depolarization Assay

In accordance with the experimental method described in previous literature,^[^ ^42^ ^]^ *S. aureus* CMCC26003, VRSA 52, and *E. coli* ATCC25922 were cultured to the logarithmic growth phase, then centrifuged at 7000 rpm and washed three times with PBS buffer. The bacterial pellet was diluted to a concentration of 5 × 10^7^ CFU mL^−1^, and 180 µL of the diluted bacterial solution was added to each well of a black 96‐well plate. DiSC_3_(5) fluorescence dyes (Sigma–Aldrich, US) were then added to the plates to achieve a final concentration of 0.5 µm. Fluorescence was recorded every 3 min using a Tecan Infinite M1000 PRO microplate reader (Tecan, Switzerland) with excitation at 622 nm and emission at 670 nm. After 21 min, drugs (vancomycin, MSI‐78, and Vm‐MSI) were added to the wells at a final concentration of 4 µm. Additionally, 20% Triton X‐100 served as a positive control, while PBS buffer was used as a negative control. The fluorescence intensity continued to be measured every 3 min after drug administration, and the experiment was stopped at 81 min to record the trend of fluorescence values.

### Antimicrobial Blockade Assay

In accordance with the experimental methods described in previous literature,^[^ ^42^ ^]^ *S. aureus* CMCC26003 and *E. coli* ATCC25922 were cultured to the logarithmic growth phase in MH medium and then diluted to a concentration of 2 × 10^5^ CFU mL^−1^. The 2 fold broth dilution method was employed to assess the changes in the MIC of the drugs (vancomycin, MSI‐78, and Vm‐MSI) in the presence of different concentrations of bacterial membrane lipids, including phosphatidylglycerol (PG, Bide Pharmatech Ltd., China), phosphatidylethanolamine (PE, Bide Pharmatech Ltd., China), cardiolipin (CL, Bide Pharmatech Ltd., China), and lipopolysaccharide (LPS, Bide Pharmatech Ltd., China).

### LPS Neutralization Assay

The LPS neutralization percentage of the coupler Vm‐MSI was assessed using the LAL Chromogenic Endotoxin Quantitation Kit (Thermo Scientific, US). According to the instruction manual, the LPS standard was dissolved in pyrogen‐free water and diluted to 5 EU mL^−1^. This solution was then added to a pyrogen‐free 96‐well plate at 50 µL per well. Concurrently, different final concentrations of the coupler solution (1, 2, 4, and 8 µm) were prepared using non‐thermal water, with 50 µL added to each well. The plate was incubated with the LPS solution at 37 °C for 1 h. After incubation, 100 µL of color development reagent was added to each well, and the plate was incubated at 37 °C for an additional 15 min. Finally, fluorescence values were recorded using a Tecan Infinite M1000 PRO microplate reader (Tecan, Switzerland) with an excitation wavelength of 380 nm and an emission wavelength of 440 nm. The LPS neutralization percentage was calculated as:

(FI_blank_‐FI_sample_)/ FI_blank_ × 100% where FI_sample_ is the fluorescence intensity of the drug incubation group and FI_blank_ is the fluorescence value of the blank control group (50 µL of heat‐free water + 50 µL of LPS solution).

### ROS Fluorescent Probe Assay

In accordance with the experimental methods described in previous literature,^[^ ^42^ ^]^ VRSA 52 and *E. coli* ATCC25922 were cultured to the logarithmic growth stage, centrifuged, and washed three times with PBS buffer before being diluted to a concentration of 1 × 10^8^ CFU mL^−1^. The DCFH‐DA probe (Sigma‐Aldrich, US) was then added at a final concentration of 10 µm, and the mixture was incubated for 30 min at 37 °C, protected from light. After incubation, the samples were centrifuged to remove residual DCFH‐DA. The stained bacterial solution was transferred to a 96‐well black plate and mixed with varying concentrations of drugs (vancomycin, MSI‐78, and Vm‐MSI). Polymyxin B was used as a positive control, while PBS buffer served as a negative control. The plates were incubated at 37 °C for 1 h, protected from light. Finally, fluorescence was recorded using a Tecan Infinite M1000 PRO microplate reader (Tecan, Switzerland) with an excitation wavelength of 488 nm and an emission wavelength of 525 nm.

### ROS‐Associated Antibacterial Assays

The experimental steps were similar to those of the ROS fluorescent probe assay, except that ROS scavengers 2,2′‐bipyridine (DP, Bide Pharmatech Ltd., China) and thiourea (TU, Bide Pharmatech Ltd., China) were added concurrently with drug administration at final concentrations of 300 mm and 80 µM, respectively. After 1 h of co‐incubation, 10 µL of the bacterial solution was aspirated and diluted 1000 fold. Subsequently, 5 µL of the diluted bacterial solution was plated on LB agar and incubated for 16 h at 37 °C. Photographs were taken of the growing colonies.

### RNA‐Sequencing and Transcriptomic Analysis

*E. coli* ATCC25922 was cultured in MH broth at 37 °C with shaking at 200 rpm until mid‐logarithmic phase. The cultures were then diluted in fresh MH medium and treated with either vancomycin (100 µg mL^−1^) or Vm‐MSI at a concentration equivalent to 1× MIC for 2 h. Following treatment, bacterial cells were collected and washed three times with PBS. After RNA extraction and quality assessment, transcriptome sequencing was performed using the Illumina HiSeq platform. Clean reads were mapped to the *E. coli* ATCC25922 reference genome, and gene expression levels were quantified using RSEM. DEGs were identified using DESeq2 with thresholds of |log_2_ fold change| ≥1 and adjusted p‐value < 0.05. GO and KEGG databases were used for functional annotation and enrichment analysis.

### qPCR Analysis of Biofilm‐Related Genes

*E. coli* ATCC25922 was cultured in MH broth to the logarithmic growth phase and subsequently diluted in fresh MH medium. Bacterial suspensions were incubated under static conditions with MH medium alone, 8 µm vancomycin, or 8 µm Vm‐MSI for 48 h. After incubation, both planktonic cells and potential biofilm‐resident bacteria were collected by gentle scraping of the well surface followed by mild sonication. Total RNA was extracted using an RNA extraction kit (Tiangen, China), and cDNA was synthesized using a reverse transcription kit (Takara, Japan). Quantitative PCR was performed using the 7500 Fast Real‐Time PCR System (Applied Biosystems, Thermo Fisher Scientific, USA) to assess the expression levels of ompR, *csgA, csgD, mlrA*, *and* rcsB. Relative expression levels were calculated by the ΔΔCt method with an internal reference gene, and results were averaged from three independent biological replicates.

### Mouse Skin Infection Model

In accordance with the experimental methods described in previous literature,^[^ ^43^ ^]^ 6‐8 weeks old female BALB/c mice weighing 18–20 g were selected for the study. Holes were punched in the backs of the mice using a hole punch, and 50 µL of vancomycin‐resistant *S. aureus* (VRSA 52) at a concentration of 1 × 10^8^ CFU mL^−1^ was added to the wounds, resulting in a localized infection model. After 6 h, 50 µL of the drugs (vancomycin, MSI‐78, and Vm‐MSI) was administered at a final concentration of 2 mg kg^−1^. Ciprofloxacin was used as a positive control, while PBS buffer served as a negative control. The drugs were administered at 24 h intervals for 3 consecutive days. Following this treatment period, drug administration was discontinued, and photographs of the wounds were taken for documentation. The area of the wounds was measured using ImageJ software. At the end of the third day, some mice were randomly selected and euthanized. The wounds were excised, ground, and homogenized, and the supernatant was collected. This supernatant was diluted 1000 fold, and 50 µL was plated on solid LB medium for colony counting. Additionally, the expression levels of inflammatory factors IL‐6 and TNF‐α in the wound supernatant were assessed using an ELISA assay (ThermoFisher, US).

### Mouse Lung Infection Model

The experimental methods employed in this study were based on previously published literature.^[^ ^64^ ^]^ 6–8 weeks old female mice weighing 18–20 g were selected for the study, and colonization was performed by administering 50 µL of a multidrug‐resistant *A. baumannii* strain (at a concentration of 1 × 10^7^ CFU/mL) via tracheal intubation into the lungs of the mice for a duration of 0.5 h. After this period, an intraperitoneal injection of the drug at a dosage of 5 mg kg^−1^ was administered. PBS served as a negative control, while ciprofloxacin was used as a positive control. After 24 h, the mice were euthanized, and their lung tissues were excised, homogenized, and diluted 10000 fold for colony counting. The lung tissue homogenate was then centrifuged to obtain the supernatant, which was further diluted 5 fold. The expression levels of inflammatory factors IL‐6 and TNF‐α in the supernatant were measured using an ELISA assay (ThermoFisher, US).

### Statistical Analysis

Data were presented as mean ± standard deviation (SD). The sample size (n) for each experiment is specified in the corresponding figure legends. Normality and homogeneity of variance were assessed prior to parametric testing, and all datasets met the assumptions required for one‐way analysis of variance (ANOVA). Statistical comparisons among multiple groups were performed using one‐way ANOVA followed by Tukey's post hoc test for pairwise comparisons. Levels of significance are denoted as follows: ^*^ *p*  <  0.05, ^**^ *p*  <  0.01, ^***^ *p*  <  0.001, and ^****^ *p*  <  0.0001. All statistical analyses were conducted using GraphPad Prism software (version 10.4.1; GraphPad Software, San Diego, CA, USA).

## Conflict of Interest

The authors declare no conflict of interest.

## Author Contributions

S.Y.L., K.W., and W.Z.S. contributed equally to this work. P.Z. and Y.P.W. conceptualized the research. Methodology was developed by S.Y.L., K.W., and W.Z.S. Investigations were conducted by X.W. and S.Y.L. Visualization of the results was performed by D.X.L. and Y.L.L. Supervision of the project was provided by Y.P.W. The original draft of the manuscript was written by K.W. and W.Z.S., while S.Y.L., K.W., W.Z.S., and Y.P.W. collaboratively worked on the review and editing of the manuscript.

## Supporting information



Supporting Information

## Data Availability

The data that support the findings of this study are available in the supplementary material of this article.
