# Supplementary material for: Vm–MSI: a Vancomycin–Antimicrobial Peptide Conjugate Combating Resistant Bacteria and Broadening the Antimicrobial Spectrum
Source: Adv Sci (Weinh). 2025 Dec 8;13(11):e03023. doi: 10.1002/advs.202503023 (PMC12931196; doi:10.1002/advs.202503023)
Supplement: Supplementary file 1 — Supporting Information [file ADVS-13-e03023-s001.docx]

Supporting Information

**Vm–MSI: A Vancomycin–Antimicrobial Peptide Conjugate Combating Resistant Bacteria and Broadening the Antimicrobial Spectrum**

*Shuangyu Li^†^, Kang Wang^†^, Wenzhuang Shi^†^, Xu Wang, Duxin Li, Yanli Liu,* *Peng Zhang^*^, Yipeng Wang^*^*

**List of Supplementary Materials**

Table S1. Sequence, origin, and mechanism of action of the four AMPs in this study.

Table S2. MIC values of vancomycin, AMPs, and conjugates.

Fig. S1. Chemical structures of intermediates and vancomycin-antimicrobial peptide conjugates.

Fig. S2. LC/MS identification and HPLC purity analysis of synthetic intermediates (Vm-SMCC) and vancomycin-antimicrobial peptide conjugates.

Fig. S3. Circular dichroism spectra of MSI-78 and Vm-MSI.

Fig. S4. Anti-biofilm activity of vancomycin and conjugates.

Fig. S5. Hemolytic activity of vancomycin and conjugates.

Fig. S6. Antibacterial rate of Vm-MSI.

Fig. S7. Anti-biofilm activity of Vm-MSI against additional bacterial strains.

Fig. S8. Anti-biofilm activity of Vm-MSI determined by TTC method.

Fig. S9. qPCR analysis of biofilm-regulatory genes in *E. coli* ATCC25922.

**Table S1. Sequence, origin and mechanism of action of the four antimicrobial peptides in this study**

| AMPs | Sequence | Source | Mechanism of action |
| --- | --- | --- | --- |
| Omiganan | ILRWPWWPWRRK-NH2 | cow | Inhibition of bacterial DNA and RNA synthesis as well as induction of membrane depolarization |
| MSI-78 | GIGKFLKKAKKFGKAFVKILKK-NH2 | frog | Disrupting bacterial cell membrane integrity |
| Pleurocidin | GWGSFFKKAAHVGKHVGKAALTHYL-NH2 | fish | Inhibition of protein synthesis as well as induction of membrane depolarization |
| Bac-7 | RRIRPRPPRLPRPRPRP | cow | Binding to the exit tunnel of bacterial ribosomes and impeding the elongation phase of protein translation |

**Table S2. MIC values of vancomycin, AMPs, and conjugates.**

| **Bacteria** | **Vancomycin** | **Vm-Omi** | **Omiganan** | **Vm-MSI** | **MSI-78** | **Vm-Ple** | **Pleurecidin** | **Vm-Bac** | **Bac-7** |
| --- | --- | --- | --- | --- | --- | --- | --- | --- | --- |
| **Gram-positive** |  |  |  |  |  |  |  |  |  |
| *S. aureus* CMCC 26003 | 0.59(0.40) | 0.78(0.22) | 2.34(1.32) | 1.17(0.28) | 2.34(0.94) | 0.78(0.17) | 1.17(0.43) | 0.78(0.20) | 37.5(17.27) |
| *S. aureus* ATCC 29213 | 4.69(3.16) | 1.17(0.33) | 18.75(10.54) | 2.34(0.55) | 37.5(15.14) | 4.69(1.05) | 9.38(3.46) | 2.34(0.59) | >100(>46.0) |
| MRSA ATCC 43300 | 4.69(3.16) | 4.69(1.32) | 37.5(21.08) | 4.69(1.10) | 4.69(1.89) | 4.69(1.05) | 2.34(0.86) | 2.34(0.59) | 9.38(4.32) |
| *S. aureus* 15772 | >100(>67.3) | >100(>28) | 18.75(10.54) | 1.17(0.28) | 18.75(7.57) | 2.34(0.52) | 9.38(3.46) | 2.34(0.59) | 75(34.53) |
| *S. aureus* 15192 | 0.59(0.40) | 0.78(0.22) | 18.75(10.54) | 2.34(0.55) | 18.75(7.57) | 2.34(0.52) | 9.38(3.46) | 1.17(0.30) | 75(34.53) |
| VRSA11 | >100(>67.3) | 75(21.13) | 37.5(21.08) | 9.38(2.21) | 37.5(15.14) | 9.38(2.10) | 4.69(1.73) | 18.75(4.76) | 75(34.53) |
| VRSA52 | >100(>67.3) | 75(21.13) | 9.38(5.27) | 4.69(1.10) | 9.38(3.79) | 9.38(2.10) | 9.38(3.46) | 18.75(4.76) | 75(34.53) |
| *E. faecalis* ATCC29212 | >100(>67.3) | >100(>28) | 75(42.15) | 18.75(4.42) | 75(30.28) | 18.75(4.19) | 4.69(1.73) | 18.75(4.76) | 18.75(8.63) |
| **Gram-negative** |  |  |  |  |  |  |  |  |  |
| *E. coli* ATCC 25922 | >100(>67.3) | >100(>28) | 18.75(10.54) | 2.34(0.55) | 4.69(1.89) | 4.69(1.05) | 2.34(0.86) | 18.75(4.76) | 18.75(8.63) |
| *A. baumannii* ATCC 19606 | >100(>67.3) | >100(>28) | 75(42.15) | 1.17(0.28) | 9.38(3.79) | 2.34(0.52) | 9.38(3.46) | 18.75(4.76) | >100(>46.0) |
| *K. pneumoniae* 9883 | >100(>67.3) | >100(>28) | >100(56.21) | 18.75(4.42) | 37.5(15.14) | 37.5(8.37) | 4.69(1.73) | >100(>25.4) | 9.38(4.32) |
| *P*. *aeruginosa* ATCC 27853 | >100(>67.3) | >100(>28) | 18.75(10.54) | 18.75(4.42) | 9.38(3.79) | >100(>22.33) | 9.38(3.46) | >100(>25.4) | >100(>46.0) |
| *S. typhimurium* ATCC14028 | >100(>67.3) | >100(>28) | 37.5(21.08) | 18.75(4.42) | 18.75(7.57) | 75(16.75) | 18.75(6.92) | 37.5(9.52) | 75(34.53) |
| **GM** | 70.04(47.14) | 65.96(18.49) | 36.00(20.23) | 8.02(1.89) | 21.82(8.81) | 20.91(4.67) | 7.30(2.69) | 26.17(6.65) | 59.14(27.22) |

MIC: Minimum Inhibitory Concentration; GM: geometric mean of MIC from tested bacterial strains, μg/ml (μM). The test was independently performed three times.





**Figure S1.** **Chemical structures of intermediates and vancomycin-antimicrobial peptide conjugates.** (**A** : Vm-SMCC, **B**: Vm-Omi, **C**: Vm-Bac, **D**: Vm-MSI, **E**: Vm-Ple)


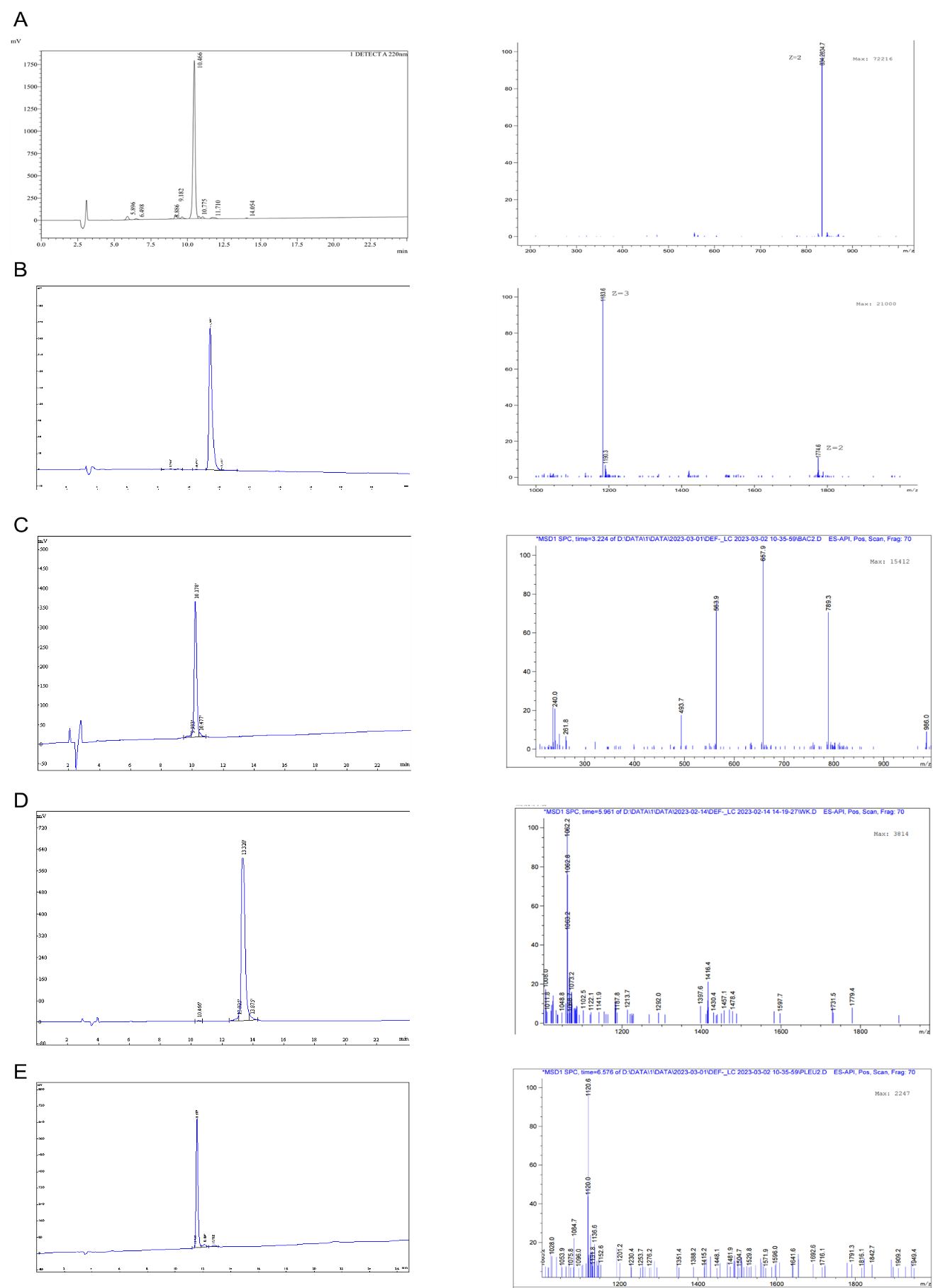


**Figure S2. LC/MS identification and HPLC purity analysis of synthetic intermediates (Vm-SMCC) and vancomycin-antimicrobial peptide conjugates.** (**A**) LC/MS identification and HPLC purity analysis of Vm-SMCC. (**B**) LC/MS identification and HPLC purity analysis of Vm-Omi. (**C**) LC/MS identification and HPLC purity analysis of Vm-Bac. (**D**) LC/MS identification and HPLC purity analysis of Vm-MSI. (**E**) LC/MS identification and HPLC purity analysis of Vm-Ple. All compounds are >95% pure by HPLC analysis.


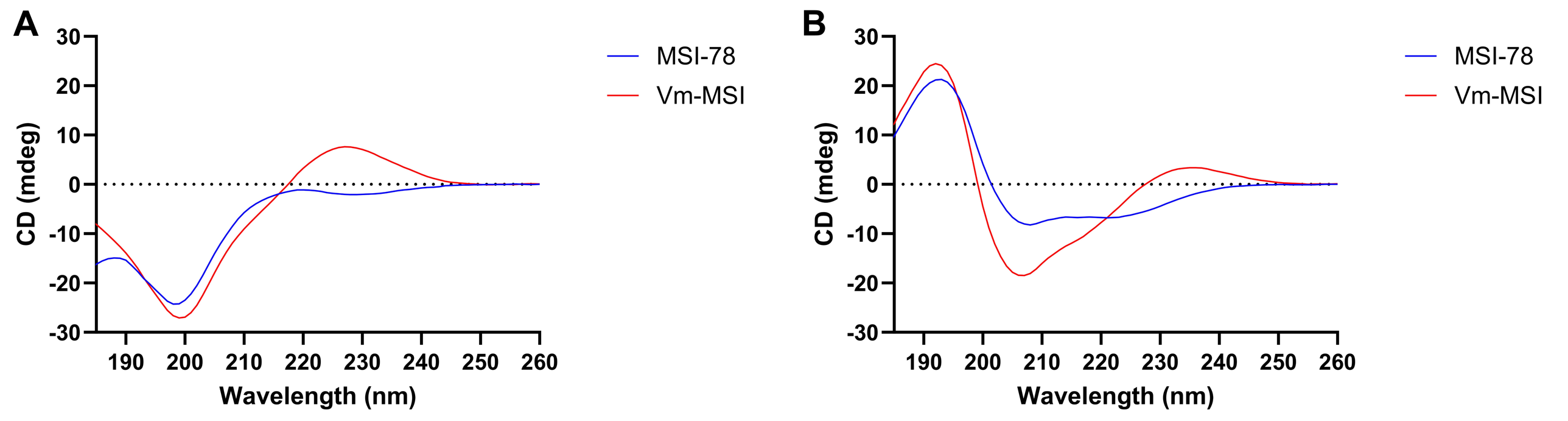


**Figure S3. Circular dichroism spectra of MSI-78 and Vm-MSI.** (**A**) CD spectra of MSI-78 and Vm-MSI in H₂O. (**B**) CD spectra of MSI-78 and Vm-MSI in 60 mM SDS solution. Each spectrum represents the median of three independent measurements. Source data are provided as a Source Data file.


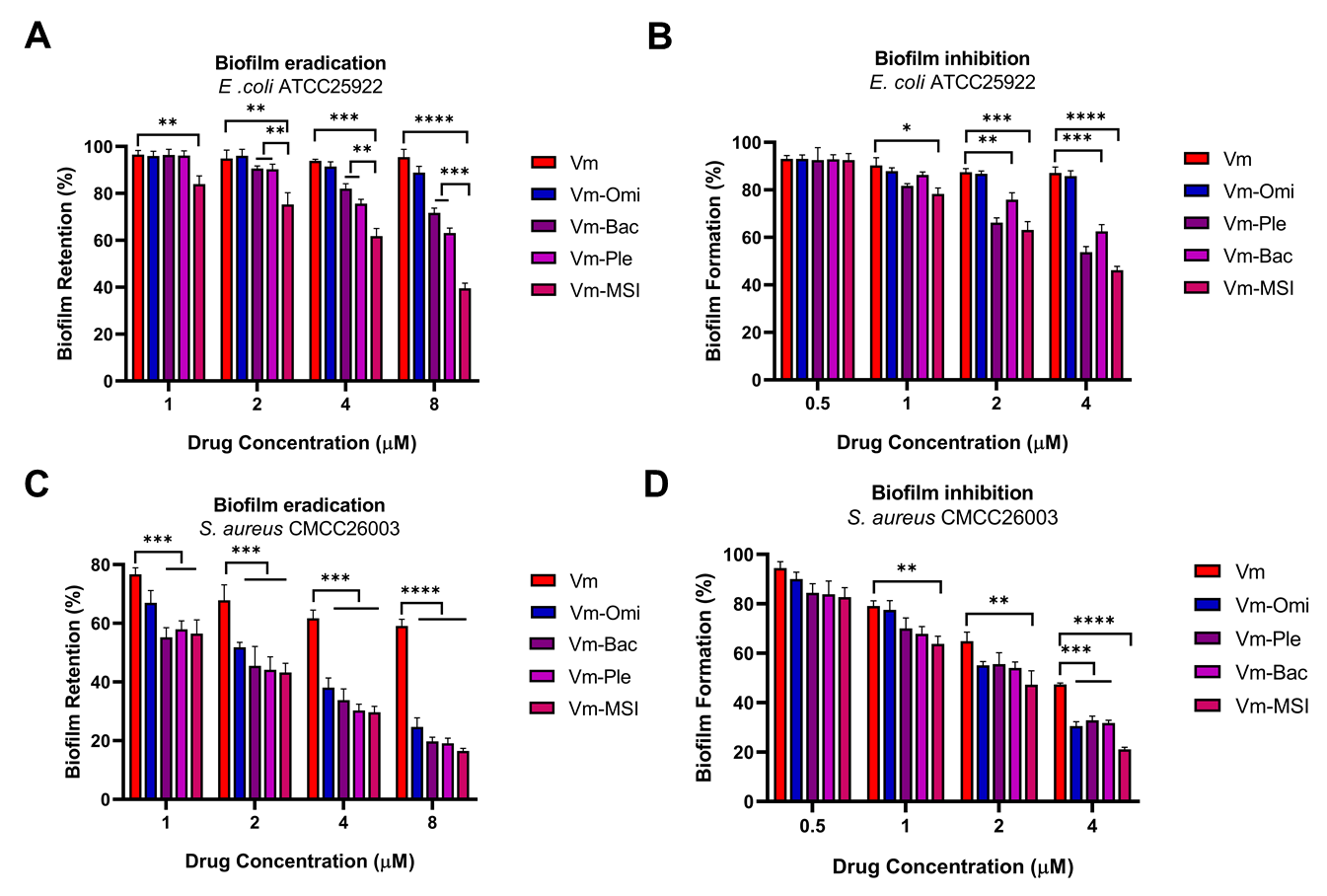


**Figure S4. Anti-biofilm activity of vancomycin and conjugates**. (**A**, **C**) Eradication activity of vancomycin and conjugates against pre-formed biofilm of *E. coli* ATCC25922 and *S. aureus* CMCC26003. (**B**, **D**) Inhibition activity of vancomycin and conjugates against biofilm formation of *E. coli* ATCC25922 and *S. aureus* CMCC26003. Each value is the median of three independent experiments. Statistical comparisons among multiple groups were performed using one-way ANOVA followed by Tukey’s post hoc test for pairwise comparisons. p**<0.01, ***p<0.001, ****p <0.0001. n = 3 biological replicates. The results are expressed as mean ± S.D. Source data are provided as a Source Data file.


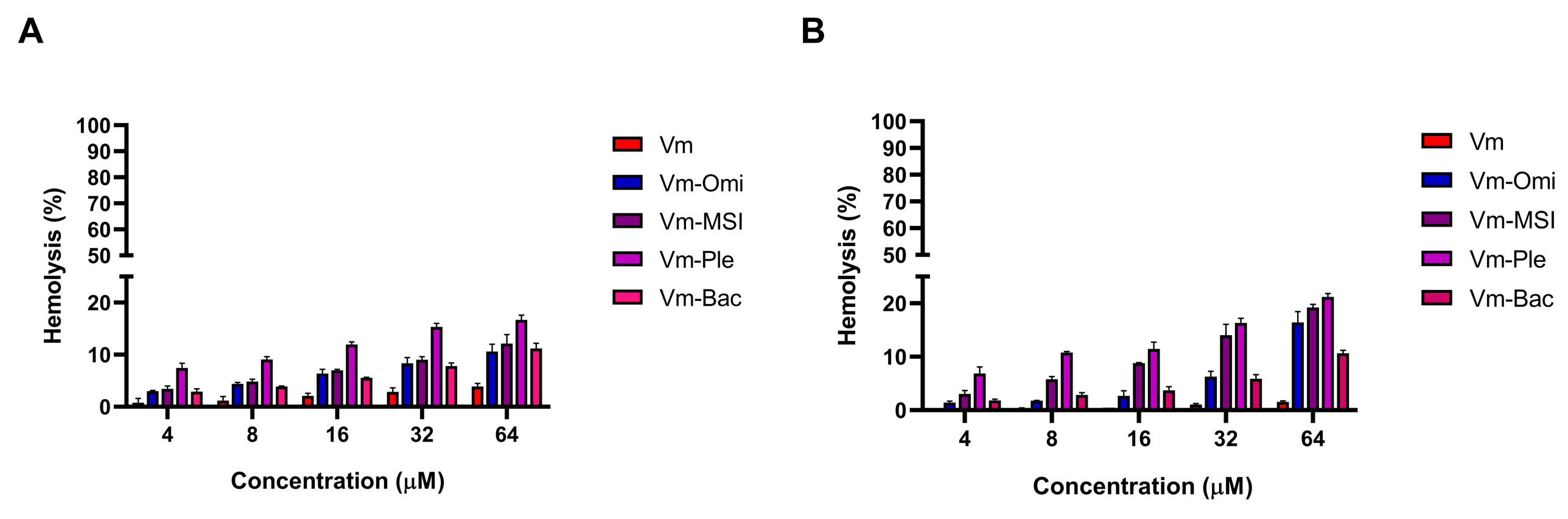


**Figure S5.** **Hemolytic activity of vancomycin and conjugates.** (**A**) Hemolysis of vancomycin and conjugates to human blood erythrocytes. (**B**) Hemolysis of vancomycin and conjugates to mouse blood erythrocytes. Each value is the median of three independent experiments. n = 3 biological replicates. The results are expressed as mean ± S.D. Source data are provided as a Source Data file.


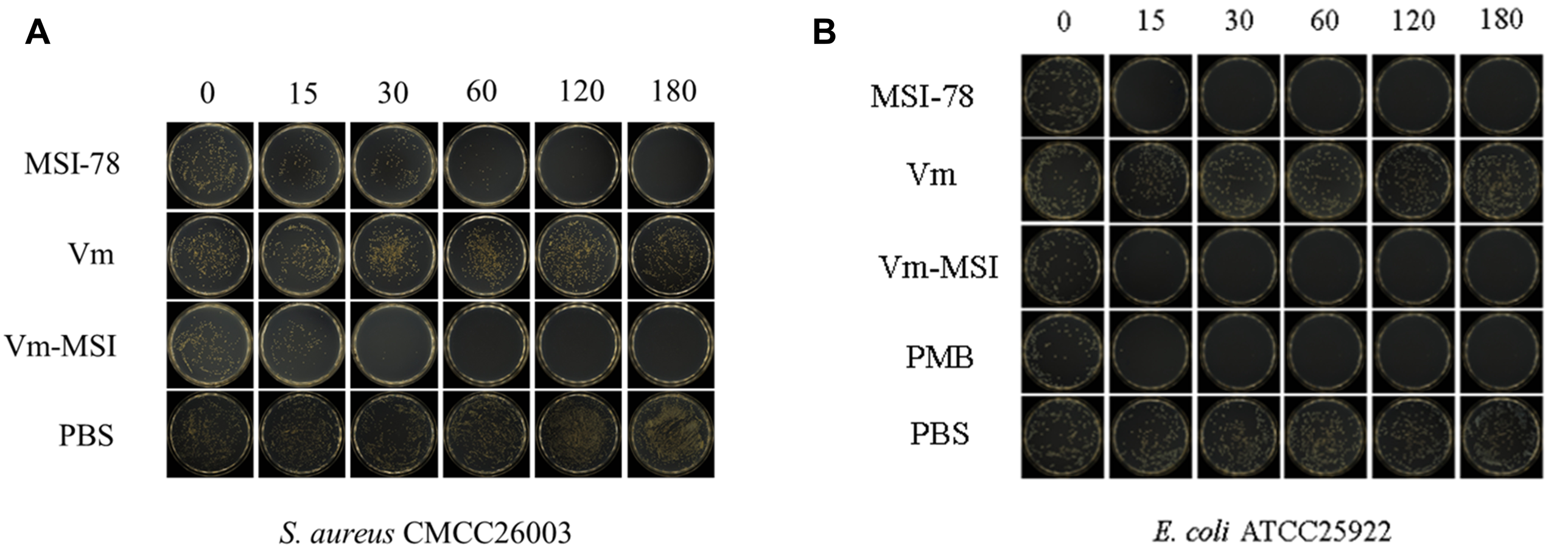


**Figure S6.** **Antibacterial rate of Vm-MSI.** 10^5^ CFU/mL of *S. aureus* CMCC26003 and *E. coli* ATCC25922 were co-incubated with vancomycin, MSI-78, or Vm-MSI (a final concentration of 4 μM) at 37°C. At time intervals of 0, 15, 30, 60, 120, and 180 minutes, 10 μL of the bacterial solution was extracted and diluted 1000-fold with PBS buffer. The diluted solution was then coated onto the surface of LB solid medium and incubated statically at 37℃ for a duration of 16 hours to observe colony formation. (**A**) *S. aureus* CMCC26003 colony counts at 0, 15, 30, 60, 120, and 180 min. (**B**) *E. coli* ATCC25922 colony counts at 0, 15, 30, 60, 120, and 180 min. The quantification of the number of colonies in this figure is presented in Fig. 2D and 2E in the main text.


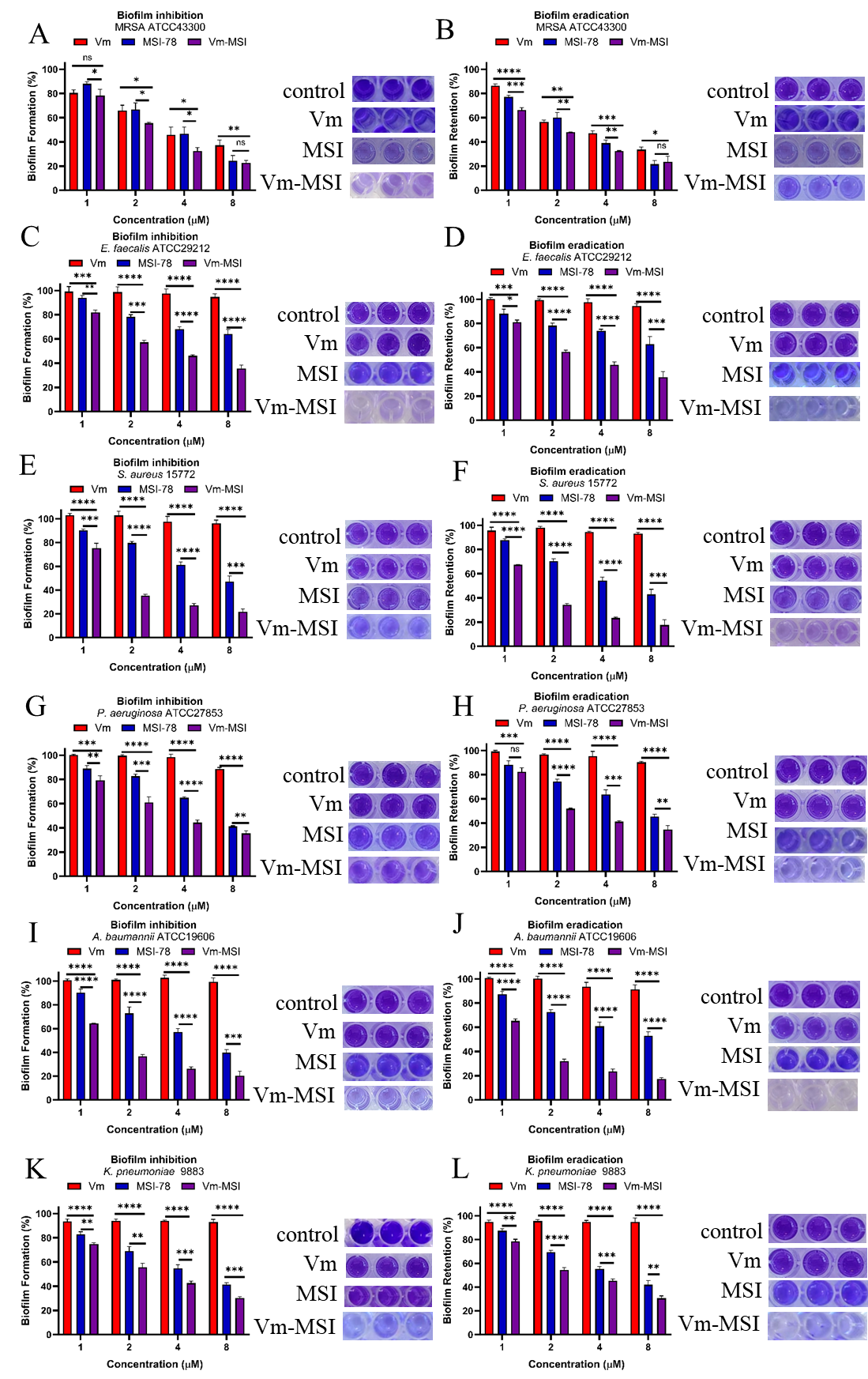


**Figure S7. Anti-biofilm activity of Vm-MSI against additional bacterial strains.** (**A, B**) Biofilm inhibition and eradication activity of vancomycin, MSI-78, and Vm-MSI against MRSA ATCC43300. (**C, D**) Biofilm inhibition and eradication activity against *E. faecalis* ATCC29212. (**E, F**) Biofilm inhibition and eradication activity against *S. aureus* 15772. (**G, H**) Biofilm inhibition and eradication activity against *P. aeruginosa* ATCC27853. (**I, J**) Biofilm inhibition and eradication activity against *A. baumannii* ATCC19606. (**K, L**) Biofilm inhibition and eradication activity against *K. pneumoniae* 9883. In the images, a darker blue-violet color indicates more residual biofilm. Each value represents the median of three independent experiments. Statistical comparisons among multiple groups were performed using one-way ANOVA followed by Tukey’s post hoc test for pairwise comparisons. p**<0.01, ***p<0.001, ****p <0.0001. n = 3 biological replicates. The results are expressed as mean ± S.D. Source data are provided as a Source Data file.


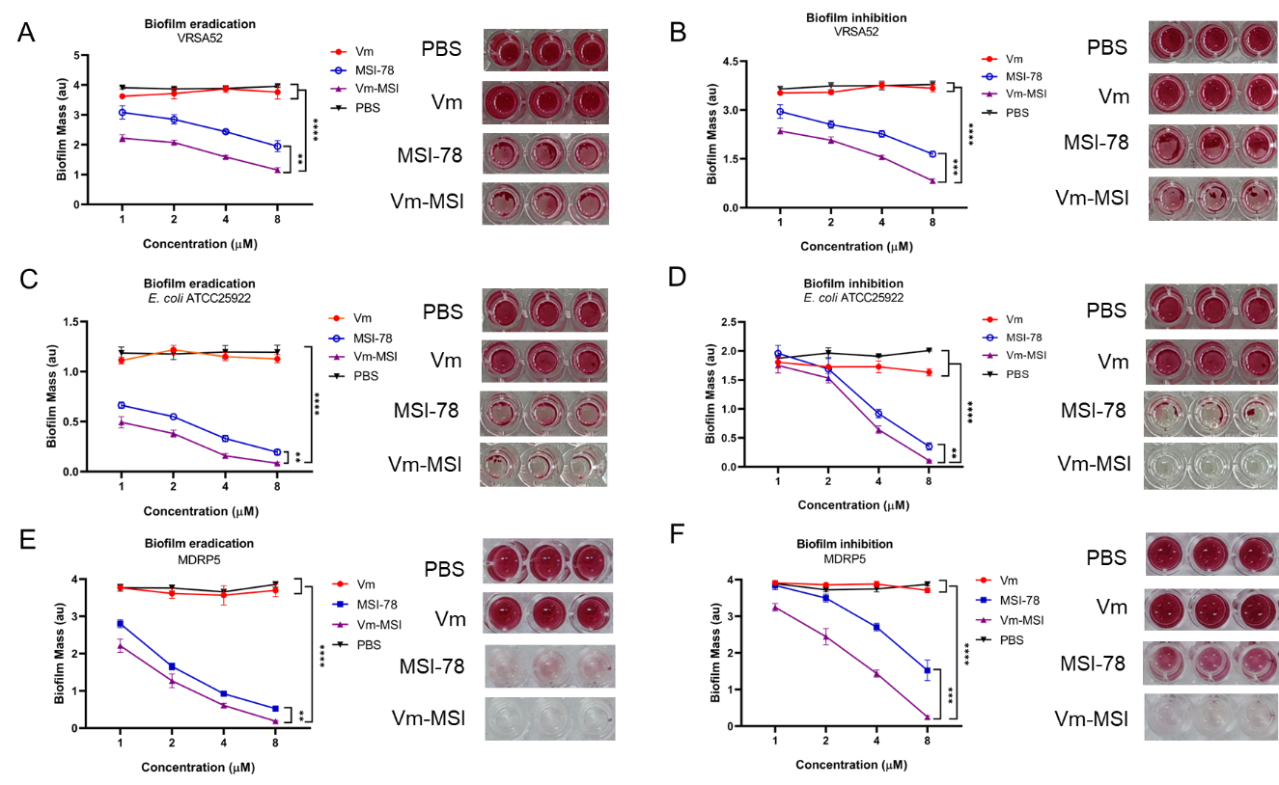


**Figure S8. Anti-biofilm activity of Vm-MSI determined by TTC method.** (**A**, **C** and **E**) Eradication ability of vancomycin, MSI-78 and Vm-MSI against pre-formed biofilms of VRSA 52, *E. coli* ATCC25922, and MDRP5. (**B**, **D** and **F**) Inhibition of VRSA 52, *E. coli* ATCC25922, and MDRP5 biofilm formation by vancomycin, MSI-78 and Vm-MSI. Each value is the median of three independent experiments. The figure on the right shows the complexes formed by the redox reaction of bacterial biofilm with TTC; The darker the color, the more biofilm stock is indicated. Statistical comparisons among multiple groups were performed using one-way ANOVA followed by Tukey’s post hoc test for pairwise comparisons. p**<0.01, ***p<0.001, ****p <0.0001. n = 3 biological replicates. The results are expressed as mean ± S.D. Source data are provided as a Source Data file.


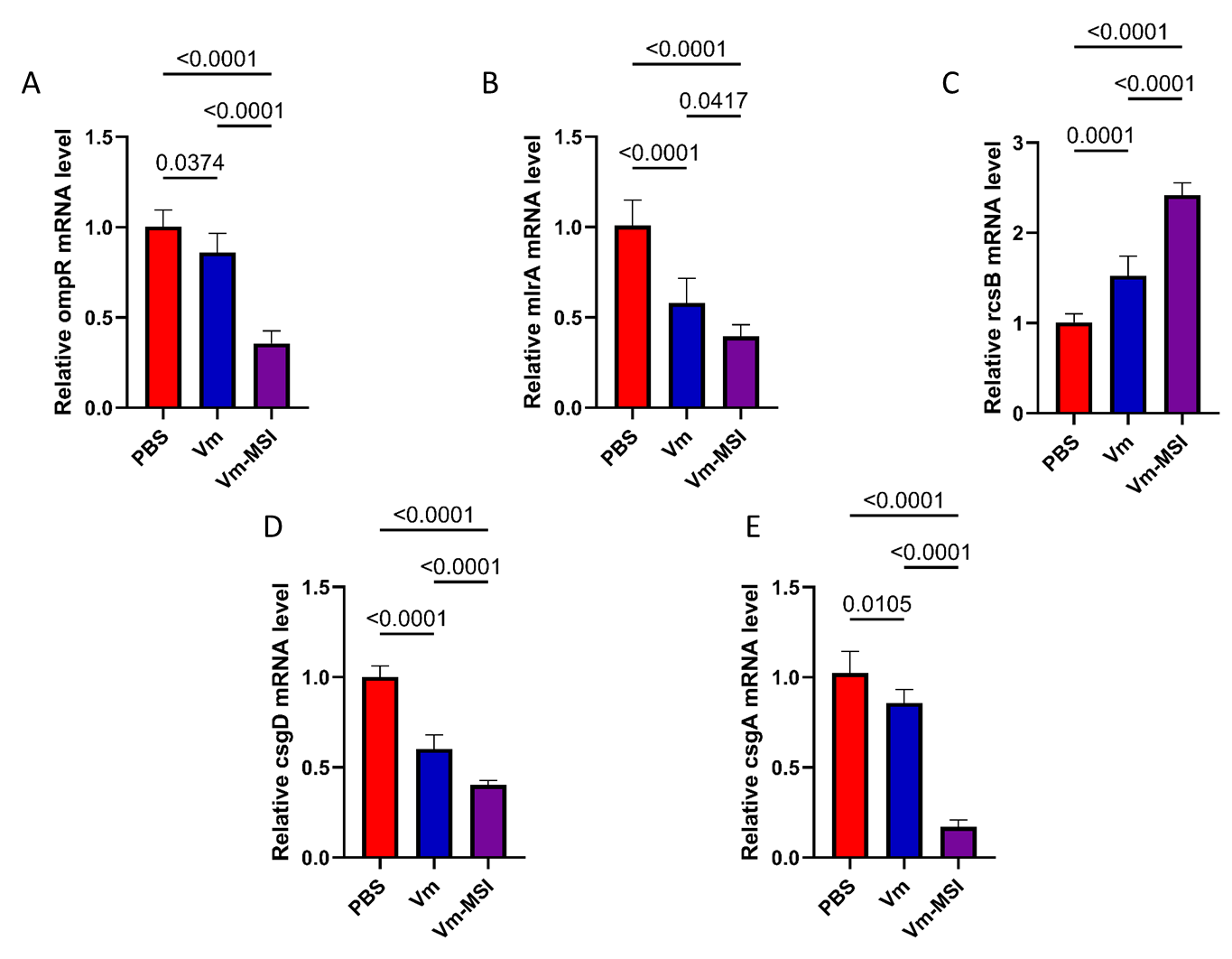


**Figure S9. qPCR analysis of biofilm-regulatory genes in *E. coli* ATCC25922.** Bacteria were incubated under static conditions with MH medium, 8 μM vancomycin, or 8 μM Vm-MSI for 48 h. Gene expression levels of (**A**) ompR, (**B**) mlrA, (**C**) rcsB, (**D**) csgD, and (**E**) csgA were determined by qPCR, normalized to the internal reference gene, and expressed as relative fold change compared with the control group. Each value represents the mean ± SD of three independent experiments. Statistical comparisons among multiple groups were performed using one-way ANOVA followed by Tukey’s post hoc test for pairwise comparisons. p < 0.05, p < 0.01, **p < 0.001, ***p < 0.0001**.** n = 6 biological replicates. Source data are provided as a Source Data file.
